# Spontaneous Oscillatory Activity in Episodic Timing: An EEG Replication Study and Its Limitations

**DOI:** 10.1523/ENEURO.0332-25.2025

**Published:** 2026-01-20

**Authors:** Raphaël Bordas, Virginie van Wassenhove

**Affiliations:** CEA/DRF/Inst. Joliot, NeuroSpin, INSERM, Cognitive Neuroimaging Unit, Université Paris-Saclay, Gif/Yvette 91191, France

**Keywords:** alpha burst, resting state, retrospective duration estimation, spontaneous alpha oscillations, time-on-task, virtual reality

## Abstract

Episodic timing refers to the one-shot, automatic encoding of temporal information in the brain, in the absence of attention to time. A previous magnetoencephalography (MEG) study showed that the relative burst time of spontaneous alpha oscillations (α) during quiet wakefulness was a selective predictor of retrospective duration estimation. This observation was interpreted as α embodying the “ticks” of an internal contextual clock. Herein, we replicate and extend these findings using electroencephalography (EEG), assess robustness to time-on-task effects, and test the generalizability in virtual reality (VR) environments. In three EEG experiments, 128 participants of either sex underwent 4 min eyes-open resting-state recordings followed by an unexpected retrospective duration estimation task. Experiment 1 tested participants before any tasks, Experiment 2 after 90 min of timing tasks, and Experiment 3 in VR environments of different sizes. We successfully replicated the original MEG findings in Experiment 1 but did not in Experiment 2. We explain the lack of replication through time-on-task effects (changes in α power and topography) and contextual changes yielding a cognitive strategy based on temporal expectation (supported by a fast passage of time). In Experiment 3, we did not find the expected duration underestimation in VR and did not replicate the correlation between α bursts and retrospective time estimates. Overall, while EEG captures the α burst marker of episodic timing, its reliability depends critically on experimental context. Our findings highlight the importance of controlling experimental context when using α bursts as a neural marker of episodic timing.

## Significance Statement

How does the brain automatically keep track of time during everyday experiences? This study investigates alpha brain activity as a marker of contextual changes during quiet wakefulness. We successfully replicated the original findings using EEG, which is more widespread than MEG, but found some limitations. This neural marker is sensitive to mental fatigue and experimental context, with participants adopting temporal expectation strategies that alter the relation between alpha and temporal estimation. Virtual reality environments may have fostered prospective timing behavior, which the alpha marker is known to be insensitive to. As alterations in timing affect numerous neurological and psychiatric conditions, establishing a robust neural marker of experiential time has important implications for both basic neuroscience and clinical applications.

## Introduction

Most of our temporal experiences are lived as one-shot moments or episodic experiences of events. The term “episodic timing” was recently used to refer to the automatic encoding of temporal information in the absence of task requirements or overt attention to time (Azizi et al., 2023). Episodic timing focuses on the neural mechanisms encoding the temporal relations between events (e.g., duration, order, speed) ultimately stored in, and constitutive of, the when of memory (Kwok et al., 2025). Neural indices of episodic timing should be indicative of participants’ subsequent duration memory, which can be probed through a retrospective estimation of elapsed time. In a recent study, participants were recorded with magnetoencephalography (MEG) during quiet wakefulness for a few minutes after which they were unexpectedly asked to report as precisely as possible how long the recording episode was. The authors reported that the increase in total alpha power (α, 8–12 Hz) during resting state was linearly predictive of participants’ retrospective time estimate. More specifically, the relative α burst time was the best predictor of retrospective duration estimation (Azizi et al., 2023). This relation vanished during prospective timing (when people knew they would have to report the duration of the recording, thus paying attention to time). α burst time was postulated to be a neural index of episodic timing.

Retrospective duration tasks are single-shot duration estimations (Chaumon et al., 2022; Balcı et al., 2023), which have been postulated to engage episodic memory processes (Michon, 1975; Hicks et al., 1976; Block, 1985). The contextual change hypothesis (Ornstein, 1969; Block, 2014; MacDonald, 2014) posits that retrospective duration estimates depend on the amount of contextual change participants experienced. Spontaneous α bursts reflect discrete shifts in brain states, which could serve as neural markers of such internal contextual changes during quiet wakefulness (Azizi et al., 2023). The fact that the relation between relative α burst time and duration was only observed retrospectively and not prospectively aligned well with this hypothesis according to which retrospective duration is reconstructed from memory, not tracked in real-time. The authors suggested that spontaneous α bursts embody the “ticks” of an internal contextual clock, which operationalized the concept of contextual change at the neural level (Azizi et al., 2023).

α oscillations (Berger, 1929) occur spontaneously (Buzsáki and Draguhn, 2004), and their functional role has been refined over time (Bazanova and Vernon, 2014). The notion of cortical idling (Pfurtscheller et al., 1996) was proposed following the seminal observation that closing the eyes increased posterior α (Adrian and Matthews, 1934). It evolved toward the functional inhibition hypothesis as a regulatory mechanism of sensory processing (von Stein et al., 2000; Klimesch et al., 2007; Jensen and Mazaheri, 2010; Jensen et al., 2012). In quiet wakefulness, fluctuations of α activity capture moment-to-moment changes of brain activity in response to external stimulations but also to internally driven cognitive processes (Sadaghiani and Kleinschmidt, 2016; Halgren et al., 2019). In internally directed thoughts such as in mind-wandering, participants are oriented toward their internal stream of thoughts rather than to external stimuli (Smallwood and Schooler, 2015). Accordingly, the dynamic changes in α fluctuations co-occur with periods of mind-wandering: for instance, α power preceding a probe is higher when participants mind-wander than when they do not (Compton et al., 2019). Mind-wandering can be interpreted as a lapse of attention, which yields to an underestimation of the duration of sensory stimuli (Terhune et al., 2017).

Herein, we wished to extend the original MEG observations. First, we wished to replicate and generalize the protocol with EEG, a widely used technique that can easily adapt to ecological situations (Stangl et al., 2023; Vallet and van Wassenhove, 2023) and clinical settings. This is important as this episodic timing marker can serve as a clinical diagnosis, seeing that time perception is altered in numerous pathologies (Hinault et al., 2023). Second, we assessed the robustness of the episodic timing marker to time-on-task effects, which are characterized by a large-scale increase in α power and/or a decrease in peak frequency (Benwell et al., 2019). Third, we tested the replicability in Virtual Reality (VR), increasingly used in time perception research (Tobin and Grondin, 2009; Tobin et al., 2010; Jording et al., 2022; Lamprou-Kokolaki et al., 2024).

In all EEG experiments, participants were recorded with open eyes in quiet wakefulness for 4 min in the absence of any task requirement; at the end of the recording, participants reported as precisely as possible the duration (in minutes, seconds) of the recording that just occurred (Azizi et al., 2023). Participants were recorded before any task (Exp. 1), after performing timing tasks for 90 min (Exp. 2), or in VR before any task (Exp. 3). We replicate the original findings when participants perform the retrospective timing task before any other task, but not after an experimental session nor in VR.

## Materials and Methods

### Participants

A total of 147 participants were recruited to take part in the study (71 males; age, 25 years old; SD = 5 years). All participants had normal or corrected-to-normal vision, were right-handed, and were naive as to the purpose of the study. None had known neurological or psychiatric disorders, and none were under medical treatment. All participants provided a written informed consent form validated by the Ethics Committee for Research (CER) of Paris-Saclay University (CER-Paris-Saclay-2018-034-A2 and CER-Paris-Saclay-2023-089) or the Research Ethics Committee of Neurospin, CEA (CPP NeuroSpin CEA 100 049 2018).

**Figure 1.**
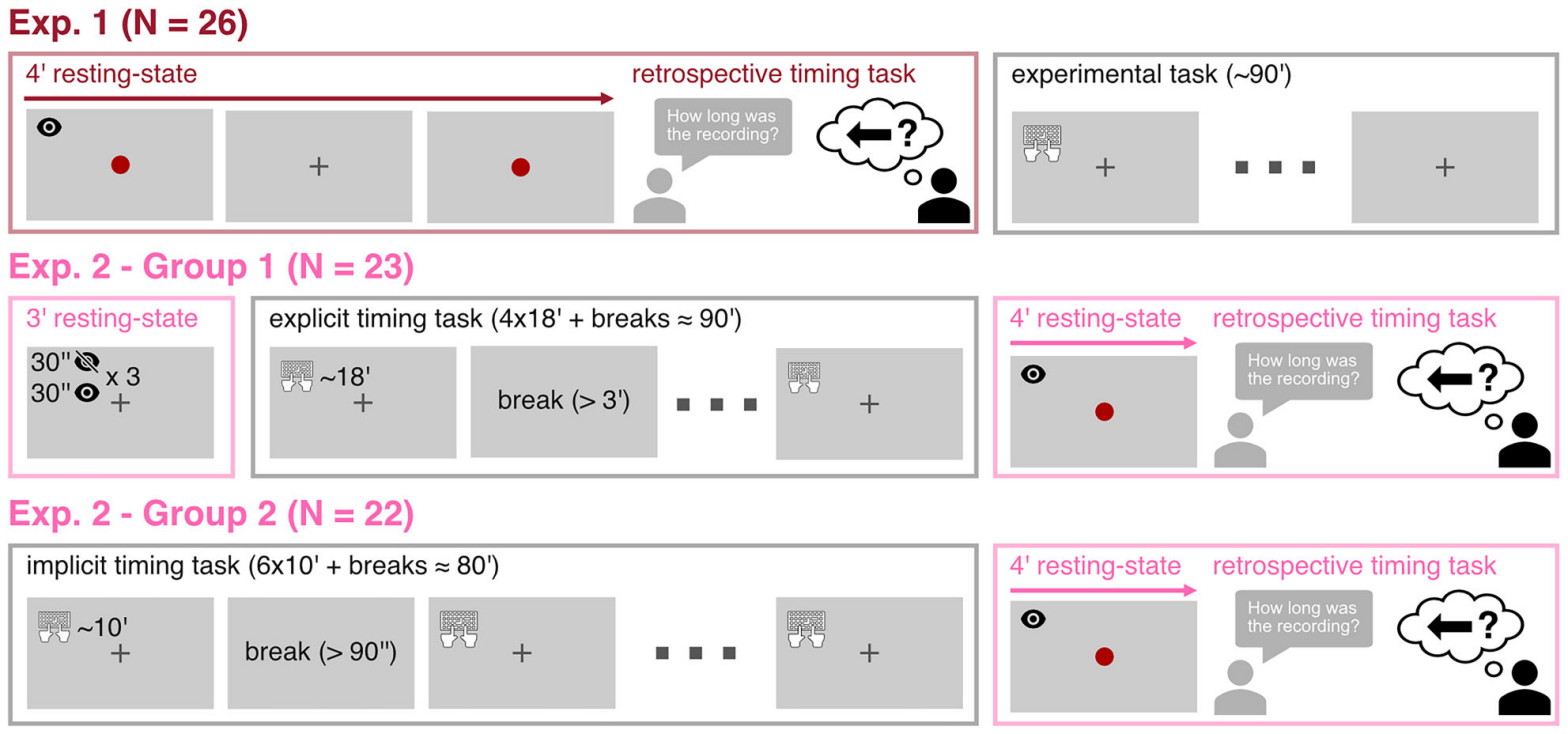
Experimental design of Exp. 1 and Exp. 2. In Exp. 1 (red frame, top panel), participants performed the retrospective timing task following a 4 min eyes-open resting state, before any other experimental task. In Exp. 2 (pink frames, middle and bottom panels), participants performed the same retrospective timing task either after an explicit timing task (*N* = 23, denoted Group 1) or after an implicit timing task (*N* = 22, denoted Group 2). Temporal structures of the experimental tasks are outlined in the gray boxes. Participants in Group 1 also performed a 3 min resting recording, alternating between eyes-open and eyes-closed states every 30 s, before the explicit timing task. Colored frames indicate which data were analyzed in this study.

Thirty-one participants took part in Experiment 1 (Exp. 1) and performed the retrospective timing task before taking part in other tasks (Fig. 1, top panel). Two participants were excluded due to inconsistent behavior, and two were excluded because they used a counting strategy against our instructions (see below). One participant was excluded because the scalp topography was outside the group distribution (Extended Data Fig. 2-1). Thus, 26 participants (15 males; age, 27 years old; SD = 5 years) were included in the final analysis of Exp. 1.

In Experiment 2 (Exp. 2), 51 participants performed the retrospective timing task ∼90 min after performing an explicit or an implicit timing task (Fig. 1, middle and bottom panels). Two participants were excluded due to technical issues in the EEG recordings, and one participant was excluded because of a counting strategy. Three additional participants were excluded because their topography was outside the group distribution (Extended Data Fig. 2-1), leaving 45 participants analyzed in Exp. 2 (20 males; age, 23 years old; SD = 5 years). Among these 45 participants, 23 were recorded during a resting-state alternating eyes-closed and eyes-open before taking part in an explicit timing task (Fig. 1, middle panel). This subset of data was analyzed to compare the α burst before and after an explicit timing task (analysis reported in Fig. 6).

In Experiment 3 (Exp. 3), 65 participants performed the retrospective timing task in a VR environment before taking part in subsequent tasks. Before doing so, and following a short VR practice session, participants filled out the Simulator Sickness Questionnaire (Kennedy et al., 1993; Bouchard et al., 2007) to ensure they were fit for the study. One participant was excluded for health reasons, two for missing data, one for bad EEG data, and one because the scalp topography was outside the group distribution. Three additional participants were excluded because they were outliers in the behavioral data distribution (see Materials and Methods). Thus, a total of 57 participants (25 males; age, 25 years old; SD = 4 years) were effectively analyzed in Exp. 3.

The required sample size was computed using an a priori power analysis of Pearson's correlation coefficients based on a published study (Azizi et al., 2023). We aimed for a power greater than 0.8, leading to a minimal sample size of 23 participants when considering a positive effect of *ρ* = 0.5 (one-sided test). The number of participants recruited in each experiment was well above the needed sample size indicated by the power analysis.

In total, 128 participants were included in the final analysis in this study (60 males; age, 25 years old; SD = 5 years).

### Experimental design

The retrospective time task consisted of an eyes-open resting-state EEG recording followed by a questionnaire as detailed in the next paragraph.

In Exp. 1 and Exp. 2, participants were seated in front of a distant wall. They were asked to stare in front of them at a point on the wall so as to attenuate eye movements, to stay as still as possible, to relax their body, and to avoid muscle and jaw tensions. They were asked to keep their eyes open and to “clear their minds”. At the end of the resting-state recording, participants were unexpectedly asked: “How much time has elapsed since the beginning of the recording? Be as precise as possible in minutes and seconds”. They were asked to report the strategy they used to make their duration estimation and to judge their felt speed of the passage of time (PoTJ) on a 5-point Likert scale ranging from 1 (Very Slow) to 5 (Very Fast). The experimenter inquired participants as to whether they were paying attention to time during the resting state or whether they guessed they would be asked to estimate the duration of the recording. None of the participants reported having done so.

In Exp. 1, participants were recorded for 4 or 5 min and performed the retrospective task before any other experimental task. In Exp. 2, participants underwent a 4 min resting-state EEG recording after completing a 90 min timing experiment, whose experimental block durations (between 10 and 18 min) and breaks (lasting at least 90 s) are outlined in Figure 1 (middle and bottom panels). The timing experiment could involve explicit attentional orientation to time using a visual temporal adaptation task Jin et al. (2024) or be an implicit timing task Herbst et al. (2022).

A subset of participants in Exp. 2 (*N* = 23) was recorded with a 3 min resting-state recording, alternating eyes open and eyes closed every 30 s before the actual explicit timing experiment. No duration estimation was asked of participants for this recording, and no indication of timing was provided. We exploited the 90 s eyes-open data for a control analysis reported in Figure 6.

In Exp. 3, participants were tested while seated in either a small or large virtual environment (Fig. 3*A*). Each resting state started with a short exploration phase of the virtual environment (i.e., 20 s). Then, participants were instructed to stay as still as possible, to clear their minds, and to stare at the lamp positioned on a table in front of them to avoid any eye movements. The start and end of the recording were indicated to the participant by the lamp, which lit up green twice: once to indicate the start of the recording and a second time to indicate the end of the recording. At the end, participants removed the VR headset and were asked the same question as in Exp. 1 and Exp. 2 and to fill out the same questionnaire as in Exp. 1 and Exp. 2.

### EEG acquisition

Forty-nine EEG datasets (26 in Exp. 1; 23 in Exp. 2) were acquired using a 32-channel EasyCap system (Brain Products) at a sampling frequency of 500 Hz, in DC (no high-pass filter) with a 131 Hz low-pass filter applied online. Impedance was kept below 25 kΩ. Seventy-nine EEG datasets in Exp. 2 and Exp. 3 were acquired with an eego mylab amplifier and a 64-channel Waveguard cap (ANT Neuro) at a sampling frequency of 1 kHz. EEG recordings were collected in DC (no high-pass filter) with an online low-pass filter of 260 Hz. The reference electrode was CPz. The vertical electrooculogram (EOG) was acquired with one electrode positioned above the left eye. Impedance was kept below 20 kΩ.

### EEG preprocessing

Data from all participants were identically preprocessed with a custom pipeline based on MNE-Python (Gramfort et al., 2013). To follow the procedure of the previous study (Azizi et al., 2023), and unless mentioned otherwise, the first 4 min of every recording were considered for analysis to homogenize the duration of the recordings across all analyses.

First, bad channels were manually marked upon visual inspection of the continuous raw signals. Mastoids were noisy and did not contain relevant brain activity (Cohen, 2014); we excluded them from further analysis. Second, we bandpass filtered all channels between 1 and 130 Hz with a notch filter set at 50 and 100 Hz to remove the power line noise and its harmonics. Third, bad channels were interpolated. Finally, data were rereferenced to the average of all channels.

To remove ocular artifacts, one copy of the signals was high-pass filtered at 1.5 Hz, downsampled to 500 Hz, and passed on to a FastICA algorithm (Gramfort et al., 2013). The classification of components containing EOG artifacts relied on a Pearson correlation between the filtered data and the filtered vertical EOG channel placed above the left eye. The automatic classification of EOG artifacts was double-checked by visual inspection through all components and manually corrected when necessary. The ICA with all the bad independent components removed was then applied to the continuous signal filtered between 1 and 130 Hz. The median number of components removed by participants was 2, with a maximum of 7.

To further ensure the quality of our data, data containing muscular artifacts were marked and rejected automatically using native MNE functions. A muscular artifact was detected when the sum of envelope magnitude across all channels divided by the square root of the number of channels in a segment exceeded a threshold set to 10 for data recorded with the EasyCap system and 15 for data recorded with the eego mylab system. To prevent fragmentation and edge artifacts of burst detections, we set the “min_length_good” parameter in mne.preprocessing.annotate_muscle_zscore to 0.5. This means that consecutive muscle artifacts separated by <500 ms were merged together, and artifacts lasting <500 ms were discarded. The remaining artifacts were marked manually and rejected during the final visual inspection. This led to up to 5 s rejected per recording in Exp. 1 (*M* = 1.70, SD = 1.64), 8 s in Exp. 2 (*M* = 1.97, SD = 2.61), and up to 28 s rejected in Exp. 3 (*M* = 4.73, SD = 5.97). Critically, to take this into account, all temporal EEG measures were relative to the recording duration after preprocessing.

### Merging datasets acquired with different systems

All recordings acquired at a sampling rate higher than 500 Hz were downsampled at 500 Hz to ensure a homogenous sample count across analyses. The intersection between montages of 64 and 32 channels was retained for all analyses, thus including 27 channels from the 10-10 international system.

### Spontaneous oscillatory spectrum and localizers

**Figure 2.**
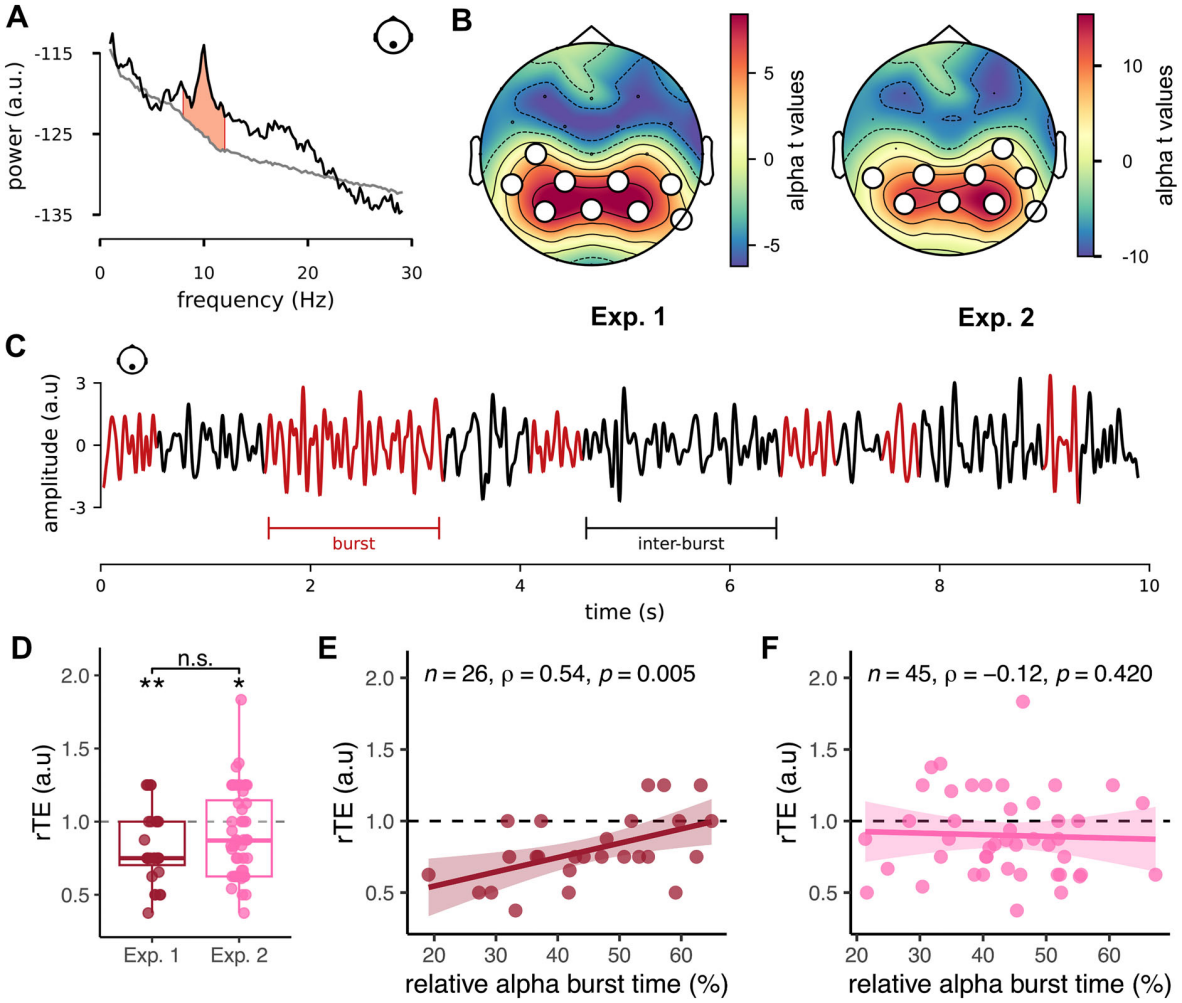
α burst time correlates positively with retrospective time estimates (rTEs) when recorded before (Exp. 1), but not after (Exp. 2), a timing task. ***A***, Spectrum decomposition using IRASA (Wen and Liu, 2016). The black line is the original spectrum computed from a single parietal channel (Pz). The gray line illustrates its aperiodic component. The red-shaded area represents the averaged power of the oscillatory spectrum between 8 and 12 Hz (α activity). ***B***, Localization of α activity in the spectral domain during a resting state acquired before (Exp. 1) or after a timing task (Exp. 2). Topographic maps in both experiments show the spatial distribution of the *t* values obtained when testing the averaged α power at each channel against the average of all channels. The white dots denote the cluster retained in all subsequent analyses and experiments. Topographies of participants that were excluded based on this localization can be found in Extended Data Figure 2-1. ***C***, Example of α bursts recorded at Pz during resting state. A burst is defined as a series of contiguous cycles with a consistent period, amplitude, and monotonicity in the α range. Each burst is shown in red. The relative burst time is the percentage of cycles classified as belonging to a burst in the entire time series. ***D***, Distribution of the relative time estimates before any task (*N* = 26; Exp. 1; red) and after performing a 90 min task (*N* = 45; Exp. 2; pink). Each dot is a participant (single-trial experiments). Standalone stars indicate a significant temporal underestimation according to a Wilcoxon signed-rank test (**p = 0.001; *p = 0.020) with the gray dotted line as the null hypothesis value (i.e., estimated duration equals veridical duration). n.s. indicates the absence of a significant difference between Exp. 1 and Exp. 2 (Wilcoxon rank-sum test, *W* = 545.50, *p* = 0.257). The homogeneity of participants grouped in Exp. 2 is assessed in Extended Data Figure 2-2. The PoTJs associated with these duration estimates are shown in Extended Data Figure 2-3. ***E***, ***F***, Spearman's correlation between the relative α burst times and the rTEs in Exp. 1 (***E***) and Exp. 2 (***F***). The straight line is the regression line, and the shaded area is the 95% CI. Sensitivity analysis of the linear model of panel ***C*** can be found in Extended Data Figure 2-4. Correlation analysis between the number of α bursts per second and the rTE can be found in Extended Data Figures 2-5, 2-6.

To detect α activity (8–12 Hz) at every channel, we used a custom Python implementation of the IRASA algorithm (Wen and Liu, 2016). IRASA is used to separate the oscillatory component from the aperiodic one (1/f slope), typically seen in the power spectral density (PSD) of brain activity (Fig. 2*A*). First, the continuous EEG signals were divided into fixed-length epochs of 5 s to allow a 0.2 Hz frequency resolution in the spectral domain. Second, the IRASA algorithm upsampled and downsampled the epoched data at pairwise noninteger scaling factors *h* and *1*/*h*. Specifically, the sampling frequency was, respectively, multiplied by *h* and *1*/*h* with *h* ranging from 1.1 to 2.95 in increments of 0.05. Third, for each (*h*, *1*/*h*) pair, the PSD was computed using Welch's periodogram between 1 and 45 Hz and the IRASA algorithm took the geometric mean of these two PSDs. Fourth, the aperiodic component was estimated from the median of all resulting PSDs. As a result, we obtained two estimations: one for the aperiodic component and one for the oscillatory component. The oscillatory spectrum resulted from the subtraction of the aperiodic component from the original PSD. The α peak power was defined as the maximum amplitude of the oscillatory spectrum between 8 and 12 Hz. The individual α peak frequency (iAPF) was defined as the argument of this maximum. The power and the iAPF of α activity were used for sanity checks in Exp. 2.

To control for broadband frequency variations, we used the slope of the aperiodic component. It was obtained via a linear regression in the log-log space, following methods from Donoghue et al. (2020). In that space, the aperiodic component $y_{ap}$ is assumed to be expressed as $y_{ap}=b-\chi \log(F)$ with F the frequency, χ the slope, and b the intercept. In our analysis, only models with *R*^2^ > 0.9 were retained to ensure the robustness of the statistics.

**Figure 3.**
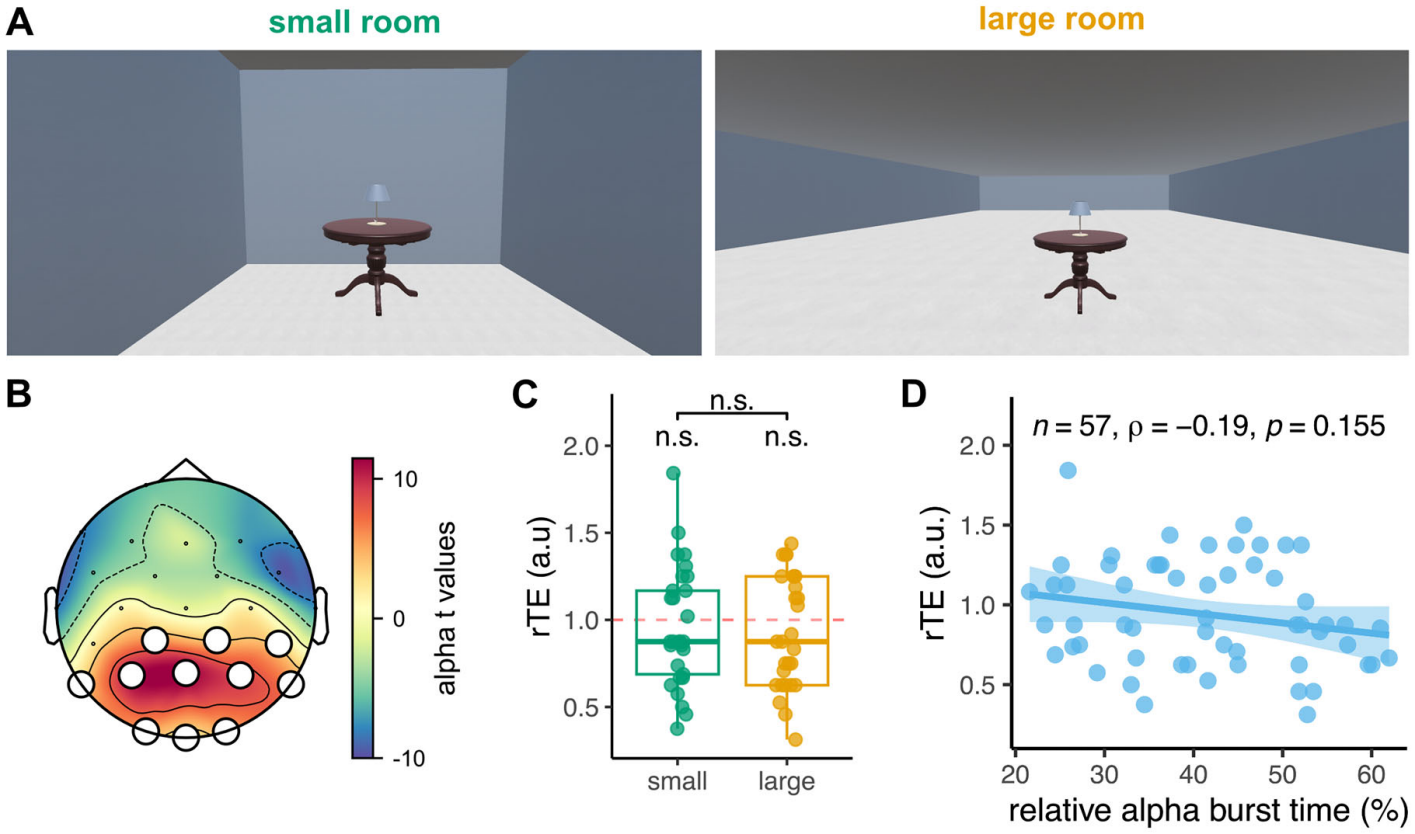
α burstiness does not predict retrospective duration in VR (Exp. 3). ***A***, Participants were seated for 4 min in a small room (*N* = 29; left panel) or a large room (*N* = 28; right panel). The lamp turned green for 500 ms at the beginning and at the end of the interval. ***B***, Localization of α activity in the spectral domain. The topographic map shows the distribution of the *t* values obtained when testing the averaged α power at each channel against the average of all channels. The white dots denote the cluster retained in all subsequent analyses. ***C***, Distribution of the rTEs in the small (*N* = 29; green) and large (*N* = 28; yellow) rooms. Each dot is a participant. No significant underestimation was found in either condition (standalone n.s. markers). No significant differences were found between experimental conditions, as assessed by a Wilcoxon rank-sum test. ***D***, Pearson's correlation between the relative α burst times and the rTEs. The straight line is the regression line, and the shaded area is the 95% CI.

Once the oscillatory spectra were obtained, we identified the most sensitive channels to the α activity. For this, a cluster-based permutation *t* test (Maris and Oostenveld, 2007) was performed on the averaged power of the oscillatory spectrum in the α range (Fig. 2*A*). Specifically, we tested whether each EEG channel had an averaged power higher than the average of all EEG channels using *t* statistics. The null distribution of this *t* test was obtained through a Monte Carlo approximation with *N* = 4,000 permutations. Using the channels connectivity matrix estimated with the Delauney triangulation implemented in MNE-Python (Gramfort et al., 2013), clusters of interest were defined as neighboring channels whose upper-tail *t* statistics exceeded a threshold *q*^(*N*−1)^. This threshold was set at the 99.9th percentile of a Student's *t* distribution with degrees of freedom *N* − 1, with *N* the number of participants. For Exp. 1, $q^{\left(26\right)}=2.48$, for Exp. 2, $q^{\left(47\right)}=2.41$, and for Exp. 3 $q^{\left(57\right)}=2.39$. The *p* value was estimated based on the proportion of permutations exceeding the observed maximum cluster-level test statistic. Only clusters with corrected *p* < 0.05 are reported. All clusters were located in the parietal channels (Fig. 2*B* for Exp. 1 and 2, and Fig. 3*B* for Exp. 3) and are referred to hereafter as “parietal clusters.” Importantly, this procedure was not used to make inferences, as we tested against a fixed value, but only to select a subset of channels. One participant in Exp. 1, three participants in Exp. 2, and one participant in Exp. 3 displayed no oscillatory activity at the group-level cluster. Hence, they were excluded from further analysis (see their profiles in Extended Data Fig. 2-1). Thus, as stated in Participants, *N* = 26 in Exp. 1, *N* = 45 in Exp. 2, and *N* = 57 in Exp. 3 were included in our final analyses (Table 1).

**Table 1.** Sample size in each experiment and for each analysis after the exclusion of participants who did not display the frequency band of interest according to the localizer analysis

	α (8–12 Hz)	θ (3–7 Hz)	β (15–30 Hz)
Exp. 1	26	24	23
Exp. 2	45	44	44
Exp. 3	57	56	54

The same procedure was separately repeated for θ (3–7 Hz) and β (15–30 Hz) activity. Table 1 summarizes the resulting sample size for each frequency band.

### Cycle-by-cycle analysis and burst detection

To compute the bursting features of continuous signals, we used the bycycle toolbox (Cole and Voytek, 2019). The bycycle algorithm computes several statistics on each cycle of a periodic time series to identify burst episodes. The settings for the detection algorithm used identical values as a previous study (Azizi et al., 2023): the amplitude fraction threshold was set to 0.2; the amplitude consistency threshold to 0.4; the period consistency threshold to 0.4, the monotonicity threshold to 0.8, and the minimum number of cycles to 3.

For the α bursts, and prior to the thresholding algorithm, the signal was bandpass filtered between 4 and 16 Hz. The bandwidth larger than the 8–12 Hz definition of α avoided strict transition bands (border artifacts). Outcomes of the bycycle algorithm were modified as follows: burst cycles with a frequency not included in (8–12 Hz) were discarded, unless they were surrounded by two burst cycles and the mean period of the burst was included in the extended α range (7.5–12.5 Hz). This procedure avoided splitting bursts due to the period variability from one cycle to another inside a burst. The outcome of this procedure was the relative burst time, which corresponds to the ratio of the number of cycles that are part of an α burst to the total number of cycles in the signal (Fig. 2*C*). As a possible interpretation of this variable, the more bursty the signal is, the more rhythmic or sustained the signal is in the time domain. On the contrary, a 0% bursting signal means that no α activity is detected.

Additionally, to assess the stability of α burstiness over time, the process described above was repeated on nine 25 s nonoverlapping time windows. This window duration took into account the muscular artifact rejection that shortened some recordings by a few seconds and ensured identical signal length for all participants (see above, EEG preprocessing).

We used the same procedure to detect bursts in the θ and β bands, with adapted thresholds and parameters. For θ (3–7 Hz), the monotonicity threshold was set to 0.7, the amplitude fraction threshold to 0.3, the minimum number of cycles to 2, and signals were prefiltered between 2 and 8 Hz. For β (15–30 Hz), the monotonicity threshold was set to 0.9, and both period and amplitude consistency thresholds to 0.5.

### Statistical analysis

All statistical analyses were performed using the R Software version 4.3.1 (R Core Team, 2021).

#### Behavioral data

Relative time estimates (rTEs) were computed as the ratio between duration estimation and clock time. Three outliers in Exp. 3 were detected when defined as values outside the interval [−1.5×IQR,1.5×IQR], where IQR is the interquartile range. No outliers were detected in Exp. 1 or Exp. 2. Because the rTEs were not normally distributed (as assessed by visual inspection of the Q-Q plots), the hypothesized underestimation of retrospective duration was assessed using a Wilcoxon signed-rank test. Likewise, rTE differences between experiments (Exp. 1 vs Exp. 2) and experimental conditions (small vs large room in Exp. 3) were analyzed using a Wilcoxon rank-sum test.

#### Regressors

Due to the non-normal distribution of the rTEs, correlations were performed using Spearman's coefficient (denoted *ρ*). The corresponding linear model was also computed to assess the robustness of the relation between each predictor and the dependent variable. Tested predictors were the relative burst time, the mean period of burst cycles, and the slope of the aperiodic component. Then, Cook's distance *D* was calculated to identify influential observations. Participants with *D* > 4 / *N* were marked as potential outliers, with *N* the total number of observations. The model was rerun without these observations to ensure no individual points drove the findings. We assessed the best predictors using AIC and the magnitude of Spearman's correlation (Extended Data Figs. 2-5, 2-6).

Ordinal logistic regressions were used for the models testing passage of time judgments using the PoTJ as the dependent variable and the experiment (Exp. 1, Exp. 2, or Exp. 3) as the regressor (Extended Data Fig. 2-3). The significance of ordinal regressions was assessed through the likelihood ratio test against the null model, which included only the intercept.

#### Sensitivity analysis

Starting with 5 participants up to 26 (our lowest achieved sample in Exp. 2) and by steps of 1, we computed the linear model $r\mathrm{TE}=b_{0}+b_{1}x$ (with x the burst time) using randomly sampled data without replacement. We repeated the process 100 times to get an average *t* value testing the coefficient $b_{1}$ against zero for each sample size (Extended Data Fig. 2-4). Significant sample sizes were defined as those with averaged *t* values greater than the 97.5% quantile of the *t* distribution.

#### Dynamics of α burstiness

We analyzed α burst dynamics using 25 s nonoverlapping windows. Pairwise *t* tests were performed to evaluate variations of the relative burst time across these windows. *p* values were corrected for multiple comparisons using Holm's method. To investigate the effect of this stability on the predictive power of the relative burst time, we computed Spearman's correlation between the relative α burst time at each window and the relative duration estimated by the participant on the entire recording (rTE). Statistical significance was assessed with a one-sided test (*ρ* > 0). Critical values for statistical significance were determined using tabulated references (Ramsey, 1989).

#### Stability of α markers before and after a timing task

A subset of participants from Exp. 2 (*N* = 23) also performed a resting-state EEG recording before performing a timing task, for a total of 90 s eyes-open and 90 s eyes-closed (Fig. 1, middle panel). To assess changes in spontaneous oscillatory dynamics before and after the task, a one-way repeated-measures ANOVA was performed on the relative α burst. The first 90 s of the retrospective duration resting-state recording was used to match durations with the prerecording resting states. Post hoc comparisons were performed using a pairwise *t* test with Holm's correction. Furthermore, to evaluate if changes in α affected channels in our cluster of interest, we ran a permutation *t* test contrasting the average relative burst time during the eyes open recording before a task and after a task. The permutation procedure mirrored the spontaneous oscillation localizer method (see above), substituting averaged power with the relative burst time differences between conditions.

## Results

### Behavioral results

Participants retrospectively estimated the duration of a 4 min resting-state EEG recording before (Exp. 1, *N* = 26) or after (Exp. 2, *N* = 45) performing a 90 min timing task or while seated in a virtual room (Exp. 3, *N* = 57). We computed the relative time estimates (rTE) as the ratio between the retrospective duration and the clock duration. An rTE of 1 signifies that participants correctly estimated the target duration, an rTE <1 that they underestimated, and an rTE >1 that they overestimated (Fig. 2*D*).

10.1523/ENEURO.0332-25.2025.f2-1 Figure 2-1 Topographic maps of excluded participants due to the lack of α activity in the cluster of channels localized by the cluster group. Download Figure 2-1, TIF file.

10.1523/ENEURO.0332-25.2025.f2-2 Figure 2-2 Exp. 2 data result from merging two homogeneous groups performing a resting-state EEG recording after a 90-minute timing task. **(A)** Behavioral distribution of Group 1 (after a temporal adaptation task) and Group 2 (after an implicit task). n.s. indicates a non-significant Wilcoxon rank-sum test (W = 226.00, p = 0.546) **(B)** Bursts distribution of Group 1 (after a temporal adaptation task) and Group 2 (after an implicit task). ns indicates a non-significant Wilcoxon rank-sum test (W = 278.00, p = 0.581). Download Figure 2-2, TIF file.

10.1523/ENEURO.0332-25.2025.f2-3 Figure 2-3 Felt passage of time judgments (PoTJ) were significantly faster in Exp. 2 than in Exp. 1 and in Exp. 3. Participants judged the experienced duration of the episodic time block on a 5-point Likert scale ranging from 1 (Very Slow, dark red) to 5 (Very Fast, dark blue). An ordinal logistic regression was computed with the PoTJ as the dependent variable and the experiment as the predictor. Significance was assessed through a likelihood ratio test against the null model (**p = 0.006; *p = 0.047). Download Figure 2-3, TIF file.

**Figure 4.**
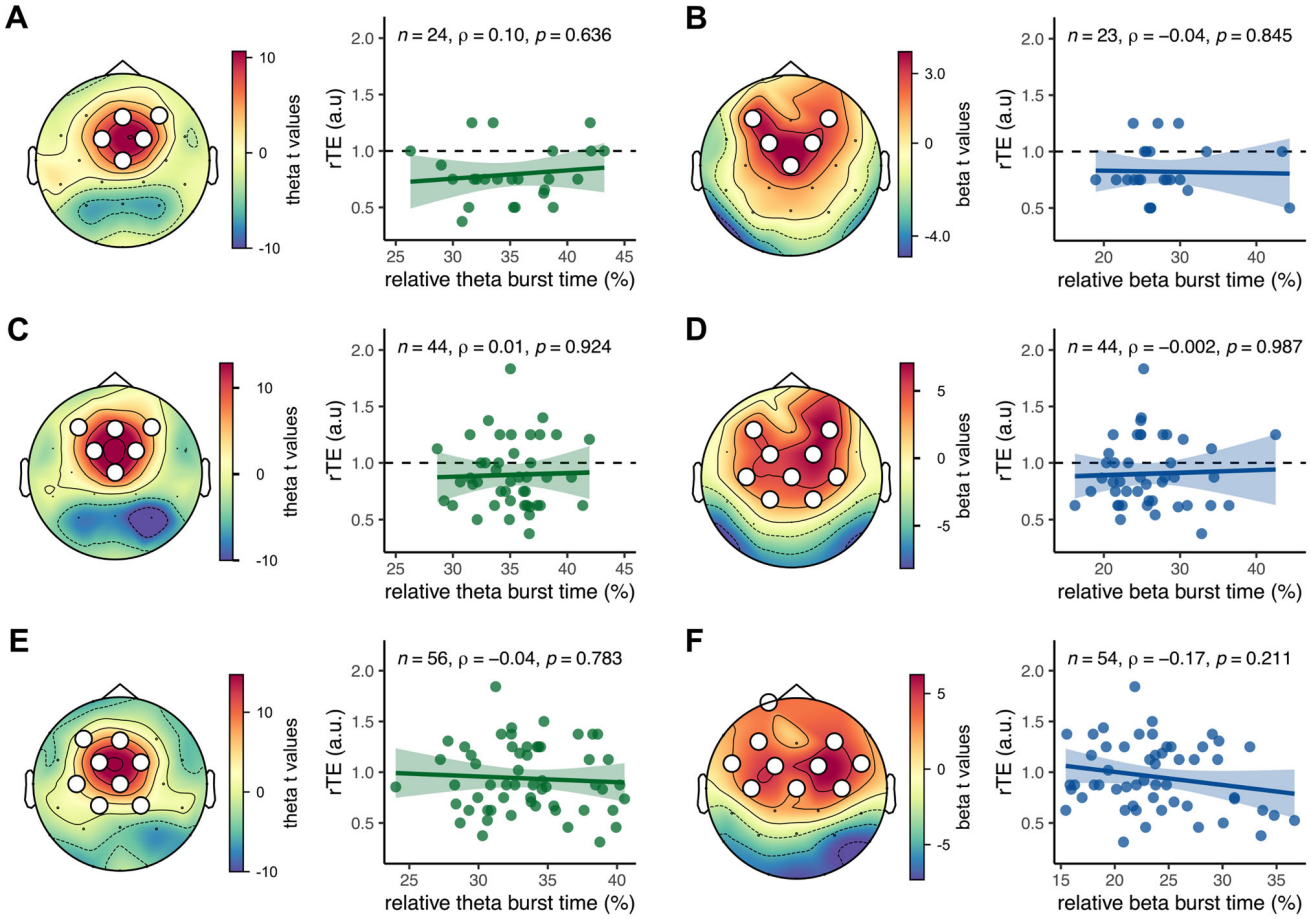
No correlation between relative θ or β burst time and rTEs. ***A***, Left, Localization of θ band activity using a permutation *t* test on the averaged power in Exp. 1. Right, Spearman's correlation between the relative θ burst time and the rTE in Exp. 1. The straight line is the regression line, and the shaded area is the 95% CI. The dotted line denotes the identity line between subjective and objective duration. ***B***, Same as ***A*** but for the β band in Exp. 1. ***C***, ***D***, Same as ***A***, ***B*** but in Exp. 2. ***E***, ***F***, Same as ***A***, ***B*** but in Exp. 3.

10.1523/ENEURO.0332-25.2025.f2-4 Figure 2-4 Sensitivity analysis of the relation between the retrospective time estimates (rTE) and the α bursts. Averaged t-value of the slope coefficient of the linear model for 100 repetitions of a sampling of n_p_ participants. n_p_ ranged from 5 to 26 (y-axis). Red dots indicate averaged t-values (over 100 repetitions) greater than the 97.5% quantile of the t distribution (with degrees of freedom of n_p_ − 2). Download Figure 2-4, TIF file.

**Figure 5.**
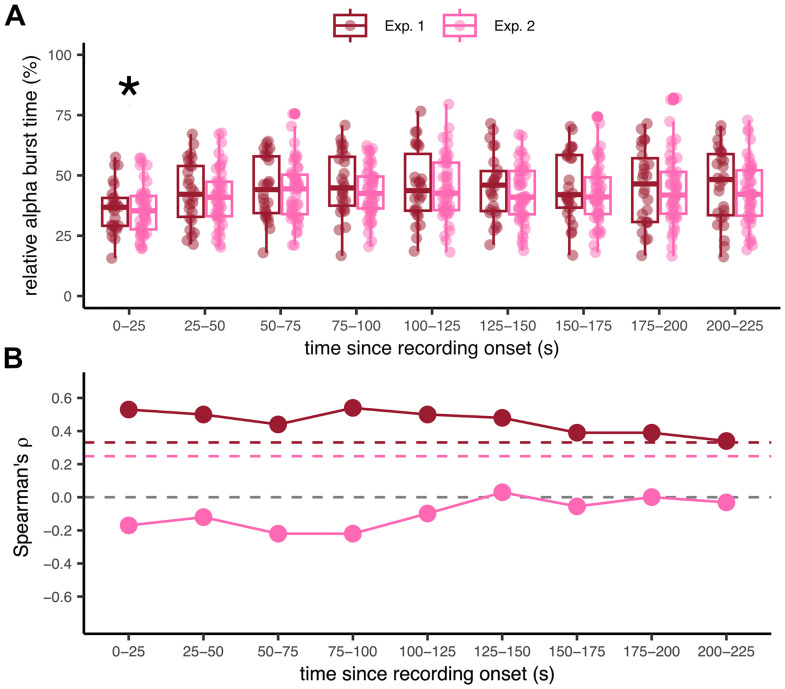
Correlation of binned α burst time and rTEs, using nonoverlapping windows of 25 s (Exp. 1 and 2). ***A***, Stability of the relative α burst time over time by windows of 25 s for Exp. 1 (red) and Exp. 2 (pink). Each dot is a participant. According to a pairwise *t* test with Holm's correction, only the first 25 s window differed significantly from all other windows in both experiments (*p < 0.002). ***B***, Spearman's correlation coefficient between the relative α burst time for each window (as shown in ***A***) and the rTE for Exp. 1 (red) and Exp. 2 (pink). The gray dotted line is the *ρ* = 0 line. For reference, the colored dotted line corresponds to the critical values of the one-sided tests (*ρ* > 0; *α* = 0.05) for *N* = 26 (Exp. 1, red line) and *N* = 45 (Exp. 2, pink line).

10.1523/ENEURO.0332-25.2025.f2-5 Figure 2-5 Spearman’s correlation between the number of α bursts per second and the rTEs in Exp. 1. The straight line is the regression line, and the shaded area is the 95% CI. Download Figure 2-5, TIF file.

**Figure 6.**
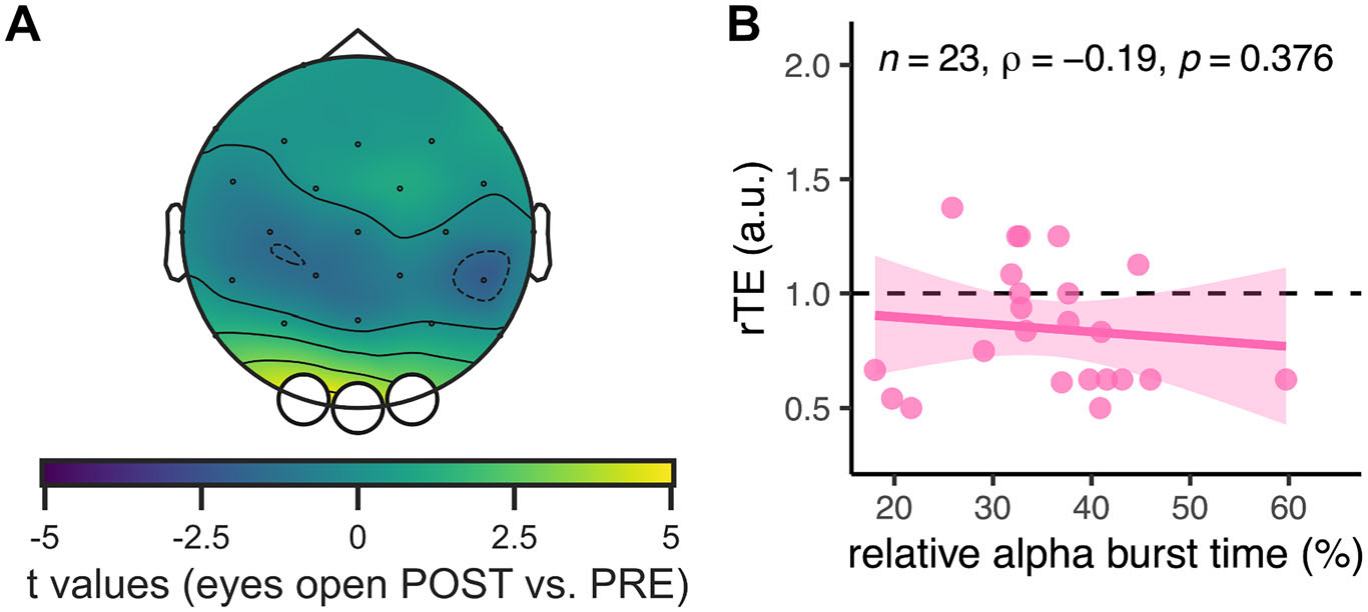
Additional contributors to α activity in Exp. 2 (*N* = 23). ***A***, Topographic map of *t* values contrasting the relative α burst time in eyes-open resting states before and after an explicit timing task in a subset group of Exp. 2 (*N* = 23). A significant cluster (white dots) was identified, showing an increase in α relative burst time. ***B***, Spearman's correlation between the relative α burst time averaged over the three channels of panel ***A*** and the rTE in the subset of Exp. 2 (*N* = 23). The straight line is the regression line, and the shaded area is the 95% CI. The dotted line denotes the identity line between subjective and objective duration.

10.1523/ENEURO.0332-25.2025.f2-6 Figure 2-6 Model comparisons for predicting the rTE in Exp. 1 (N = 26). The ⍴ denotes Spearman's correlation coefficient. The F-statistic assesses whether the regression model to predict the rTE is better than a constant value, along with the corresponding p-value of the F-test. R^2^ represents the coefficient of determination, and lower values of the Akaike Information Criterion (AIC) indicate a better fit. Bold values indicate the best models, as determined by Spearman’s correlation and AIC. Note that the discrepancy of the best predictors according to Spearman’s correlation (non-parametric measures) and AIC (based on parametric models) can be explained by the differing robustness of the predictors: the relative burst time is more robust, whereas the number of bursts per second is more prone to segmentation artefacts (e.g., splitting a burst) depending on the threshold values described in the Methods section. Download Figure 2-6, DOCX file.

On average and as predicted, participants in Exp. 1 significantly underestimated the duration of the recording (*M* = 0.80, SD = 0.24), as confirmed by a Wilcoxon signed-rank test (*W* = 24.00, *p* = 0.001).

Participants in Exp. 2 either performed a temporal adaptation task (*N* = 23) or an implicit timing task (*N* = 22) before the resting state constituting the retrospective duration to be estimated. Before merging these two datasets, we confirmed that there were no behavioral differences between these two groups of participants (*W* = 226.00, *p* = 0.546; Extended Data Fig. 2-2). In Exp. 2, like in Exp. 1, participants significantly underestimated the duration of the recording (*M* = 0.90, SD = 0.30; *W* = 251.50, *p* = 0.020; Fig. 2*D*). The observed underestimation of elapsed duration suggests that participants were unlikely to be paying attention to time during the resting-state recordings.

Contrary to Exp. 1 and Exp. 2, we found three outliers in the distribution of duration estimates in Exp. 3. All outliers overestimated durations (see Materials and Methods). There were no significant underestimations of elapsed duration in Exp. 3, when participants performed the resting-state recording in either virtual rooms (*M* = 0.94, SD = 0.33, *W* = 654, *p* = 0.171). Furthermore, we found no significant differences in the duration estimates between participants seated in the small VR (*N* = 29) and those seated in the large VR (*N* = 28) (*W* = 419.5, *p* = 0.835; Fig. 3*C*).

In addition to estimating durations, participants were also asked to judge their felt speed of the passage of time on a 5-point Likert Scale, ranging from “Very Slow” to “Very Fast.” We conducted an ordinal logistic regression with the PoTJ as the dependent variable and the experiment as the predictor (Extended Data Fig. 2-3). The model has a significantly better fit than the null model (*χ*^2^_(2)_ = 10.46, *p* = 0.005). At the predictor level, the study type was a significant predictor (Exp. 2 vs Exp. 1: *β* = 1.44, SE = 0.47, *z* = −3.07, *p* = 0.006, Exp. 2 vs Exp. 3: *β* = 0.892, SE = 0.39, *z* = 2.266, *p* = 0.047), showing that participants in Exp. 2 rated their felt passage of time to be faster than participants in Exp. 1 and in Exp. 3 (Extended Data Fig. 2-3). This observation will be important to account for the EEG observations in Exp. 2. The felt passage of time in Exp. 1 and Exp. 3 did not significantly differ (Extended Data Fig. 2-3).

### EEG replication: α burstiness correlates with retrospective duration estimates

Using the cluster of parietal channels obtained with the α localizer, we averaged the relative α burst time over these channels to get one data point per participant in Exp. 1. We computed the linear model with α burst time as the main predictor of the rTE, which was statistically significant (*R*^2^ = 0.27, *F*_(1,24)_ = 8.86, *p* = 0.007). A significant positive relation between α burst time and rTE was found (*β* = 0.01, 95% CI [3.06 × 10^−3^, 0.02], *t*_(24)_ = 2.98, *p* = 0.007). Seeing that the correlational quantification of relative α burst time and rTE entails a single measure per participant, extreme interindividual variability could disproportionately skew this relation. One participant was considered influential in this model (Cook's distance returned *D* = 0.25 with *D* > 4 / *N* = 0.15) but the removal of this data point from the model did not affect the outcomes (*β* = 0.01, 95% CI [5.16 × 10^−3^, 0.02], *t*_(23)_ = 3.71, *p* = 0.001). We thus confirmed the relation between α burst time and rTE using Spearman's correlation coefficient on the entire dataset (*ρ*_(24)_ = 0.54, *p* = 0.005; Fig. 2*E*).

To assess the reliability of our observations, we asked how many participants would be required to conclude on the presence of an effect. The sensitivity analysis of the linear model showed that a minimum of 14 participants was necessary to detect the effect in Exp. 1 (Extended Data Fig. 2-4). For this sample size, the *t* threshold equated to 2.18, and the average observed *t* value across the 100 repetitions was 2.23.

In sum, the observation of a significant positive correlation between relative α burst time and rTE observed in Exp. 1 replicated the original MEG findings (Azizi et al., 2023) and extended them to EEG. We then turned to Exp. 2, in which data were collected at the end of an experimental session.

### α burstiness does not predict retrospective duration after timing tasks

Prior to merging the two groups of participants from Exp. 2, we checked that the relative α burst times were comparable in the two groups. Using a Wilcoxon rank-sum test, we found no significant differences in relative α burst time between the two groups of participants (*W* = 278.00, *p* = 0.581). Data from all participants were thus merged into a single experimental group.

We investigated the relationship between relative α burst time and rTE in Exp. 2 using a similar approach as the one used in Exp. 1. The linear model with α burst time as the main predictor of the rTE was not significant (*R*^2^ = 0.002, *F*_(1,43)_ = 0.07, *p* = 0.788). Two participants were influential observations in Cook's sense, and the removal of these two data points did not change the outcome of the model (*R*^2^ = 0.004, *F*_(1,41)_ = 0.18, *p* = 0.675). Overall, we found no significant correlation between rTE and relative α burst time in Exp. 2 (*ρ*_(43)_ = −0.12, *p* = 0.420; Fig. 2*F*). We also performed the analysis separately for each group but found no significant correlations, whether participants took part in an explicit temporal adaptation task (*ρ*_(21)_ = −0.18, *p* = 0.420) or in an implicit timing foreperiod task (*ρ*_(20)_ = −0.11, *p* = 0.635) before the quiet wakefulness of the retrospective duration task.

### α burstiness does not predict retrospective duration in a VR environment

In Exp. 3, 57 participants waited for 4 min in either a small or a large virtual room (Fig. 3*A*, balanced sample). As for Exp. 2, we checked that the relative α burst times were comparable in the two experimental conditions. First, we averaged the relative burst time over the channels of the parietal cluster (Fig. 3*B*), which was comparable with the one found in Exp. 1 and Exp. 2 (Fig. 2*B*) with a few more occipital sensors. Then, using an unpaired *t* test, we found no significant differences in relative α burst time between the two groups of participants (*t* = 0.162, *p* = 0.872).

Additionally, we checked that the relative α burst times were comparable with Exp. 1, in which participants also performed the resting-state recording before any task. We found no significant differences between the two groups of participants (*t* = 0.96, *p* = 0.158).

Last, we investigated the relationship between relative α burst time using similar approaches as the one used in Exp. 1 and Exp. 2. The linear model with α burst time as the main predictor was not significant (*R*^2^ = 0.05, *F*_(1,55)_ = 2.74, *p* = 0.104). No participants were influential observations in Cook's sense. Overall, we found no significant relation between rTE and the relative α burst time in Exp. 3 (*ρ*_(55)_ = −0.19, *p* = 0.155; Fig. 3*D*).

### Specificity of α burstiness for the prediction of retrospective duration estimates

α burst dynamics may be a selective predictor of rTEs if no other part of the oscillatory spectrum predicts rTEs. To verify this, we performed the same correlation analysis as we did with α but this time, using slower (θ) or faster oscillations (β) known to present bursty dynamics (Jones, 2016; Little et al., 2019). Both θ and β were detected through a localizer approach, as was done for the α (see Materials and Methods).

θ bursts were located in frontal channels and θ burst time showed no correlation with the rTE in either experiment (Exp. 1 *ρ*_(22)_ = 0.10, *p* = 0.636 in Fig. 4*A*; Exp. 2 *ρ*_(42)_ = 0.01, *p* = 0.924 in Fig. 4*C*; Exp. 3 *ρ*_(54)_ = −0.04, *p* = 0.783 in Fig. 4*E*).

Similarly, β burst activity clustered in a larger set of frontal channels, and no significant correlations were found between relative β burst time and rTE (Exp. 1 *ρ*_(21)_ = −0.04, *p* = 0.845 in Fig. 4*B*; Exp. 2 *ρ*_(42)_ = −0.002, *p* = 0.987 in Fig. 4*D*; Exp. 3 *ρ*_(52)_ = −0.17, *p* = −0.211 in Fig. 4*F*).

### The dynamics of α activity are stable during resting state

We then investigated putative differences in electrophysiological data in the course of the experiment to understand why post-task α could not predict retrospective duration. Azizi et al. (2023) found that the first 2 min, and even the first 30 s, of the MEG signal was sufficient to predict participant retrospective duration estimates. This effect was explained by the stability of the α burst time over their resting-state recordings. Thus, we checked whether the α burst time recorded with EEG was stable in Exp. 1 and in Exp. 2 and compared them for eventual differences, which could account for the absence of replication in Exp. 2.

First, we tested whether relative α burst time changed over time (Fig. 5*A*). A pairwise *t* test with Holm's correction for multicomparisons revealed that only the first window (0–25 s) had a significantly lower burst time than all other windows in both Exp. 1 (*t*_(25)_ ≤ −3.59, *p* < 0.006) and Exp. 2 (*t*_(44) _≤ −3.91, *p* < 0.001). A Spearman's correlation test between the relative α burst time in each window and the participants’ rTE on the whole recording revealed a significant positive correlation in Exp. 1 (0.34 ≤ *ρ*_(24)_ ≤ 0.54; Fig. 5*B*, red line). All coefficients exceeded the critical value *c* = 0.331 corresponding to the one-sided test [*ρ* > 0; *α* = 0.05; *N* = 26; Ramsey (1989) ]. On the contrary, no correlations were found in Exp. 2 (−0.22 ≤ *ρ*_(43)_ ≤ 0.03; Fig. 5*B*, pink line) seeing that all coefficients were inferior to the critical value *c* = 0.248 corresponding to the one-sided test (*ρ* > 0; *α* = 0.05 and *N* = 45). Last, and similar to the observation in Azizi et al. (2023), the correlation coefficients in Exp. 1 slightly decreased over time, while remaining significant all along.

### Modulation of α activity over time and time-on-task effects

Time-on-task effects are systematic behavioral and neurophysiological changes that accompany the time spent on a given task. Recent work has described that time-on-task effects were associated with an increase in α power and a decrease in individual α peak frequency (iAPF) in the course of an experiment (Benwell et al., 2019). Herein, we investigated whether the lack of replication in Exp. 2 resulted from time-on-task effects in the α activity.

In Exp. 2, we also made 90 s resting-state measurements at the beginning of the recording session, which allowed assessing intraindividual changes in the evolution of α throughout the experimental session. To directly test for the time-on-task effects and their possible associated changes in α dynamics, we quantified α power and iAPF in the resting states collected before and after the experimental session. The power of the α peak was significantly higher after the experimental session (*M* = 21.6, SD = 11.2) than it was before (*M* = 18.7, SD = 11.7), as assessed by a paired *t* test (*t*_(22)_ = 2.551, *p* = 0.018). No significant changes were found in the iAPF recorded before (*M* = 10.30, SD = 0.46) and after a timing task (*M* = 10.20, SD = 0.43; *t*_(22)_ = −0.967, *p* = 0.344). Hence, we partially found time-on-task effects associated with α changes of power but not frequency.

Next, we asked whether the increased α power was caused by changes in relative burst time, seeing that two characteristics of α burst activity can cause an increase in spectral power: the average burst amplitude and the relative burst time (Feingold et al., 2015; Jones, 2016; Donoghue et al., 2022; Azizi et al., 2023). First, we quantified possible changes in burst amplitude before and after the experimental session. For this, we used a Wilcoxon signed-rank test for the contrast because the distribution of the burst amplitude recorded before the tasks did not follow a normal distribution (Shapiro's test; *W* = 0.86, *p* = 0.001). Contrasting the average burst amplitude over the parietal cluster (Fig. 2*B*), we found that the burst amplitude was significantly larger after (*M* = 9.64 µV, SD = 3.75 µV) than before an experimental session (*M* = 9.02 µV, SD = 4.09 µV; *W* = 42, *p* = 0.002). Second, we compared the average relative burst time in the parietal cluster before (*M* = 40.42, SD = 10.40) and after (*M* = 41.38, SD = 10.74) the experimental session using a paired *t* test. We found no significant differences (*t*_(22)_ = −0.79, *p* = 0.440; Cohen's *d* for paired samples of *d* = −0.16). However, when contrasting the relative α burst time before and after the experimental session, we found a significant cluster of three channels in the occipital area (Fig. 6*A*). None of these three channels overlapped with the original localizer cluster for α (Fig. 2*B*). The relative α burst time over this three-channel cluster did not correlate with the rTE either (*ρ*_(21)_ = −0.19, *p* = 0.376; Fig. 6*B*). Taken altogether, these observations suggest that α activity is partially affected by time-on-task effects and that there are additional contributors to the generation of α activity recorded at the scalp level at the end of experimental sessions.

## Discussion

In this series of EEG recordings, we replicate the original MEG observation reporting a positive linear correlation between resting-state α burst time and retrospective time estimation (Azizi et al., 2023). This replication suggests that the generators of α contributing to episodic timing can be captured noninvasively with both neuroimaging techniques. As in the initial report, this correlation was selective to α activity, and no such correlations were found for θ or β burst times during resting state, despite their clear localization in the EEG spectrum and topographies. Intriguingly however, the replication only held when resting-state and retrospective timing estimates were collected before any tasks but disappeared when collected after participants performed timing tasks for ∼90 min. Additionally, we failed to replicate the correlation between α burst time and rTE when participants were seated in VR before any task. We discuss below the diverse reasons for replication and nonreplication in this study.

### α dynamics recorded with EEG and MEG

One aim of the study was to replicate and generalize prior MEG observations to EEG. EEG remains a cost-effective technique and an invaluable tool for clinical applications and real-life mobile experimentation (Gramann et al., 2014; Stangl et al., 2023). Although MEG and EEG are both noninvasive and millisecond-resolved neuroimaging techniques, they differ in signal properties. MEG records the magnetic component of neural currents, which is less distorted by volume conduction through the dura and skull layers than the EEG signals (Baillet, 2017). This typically yields a higher signal-to-noise ratio in MEG, allowing experimental effects to be observed at the scale of an individual participant, whereas EEG tends to require population averaging. The α episodic marker investigated herein is an interindividual correlational measure that could be highly sensitive to these differences in signal-to-noise ratio. With results of Exp. 1, we nevertheless replicate the original observations seen in MEG suggesting that the signal-to-noise of EEG is sufficient even with a reasonably low sample size (i.e., below 30 participants).

The sensitivity profiles between MEG and EEG also differ: EEG can theoretically detect sources in any orientation, whereas MEG is less sensitive to radial sources (Ahlfors et al., 2010). EEG tends to be more sensitive to neural generators located in gyri and MEG to sulci. As the relation between α burst time and retrospective time holds despite these theoretical differences, this suggests that the neural sources contributing to the elicitation of spontaneous α bursts are either mostly radially oriented or largely distributed across cortex.

The sources of spontaneous α activity recorded with MEG and EEG are typically seen in occipital and parietal sensors, and source-localized in occipitoparietal regions (Salmelin and Hari, 1994; Haegens et al., 2014; Azizi et al., 2023, Extended Data Fig. 1-2). Still, this is a coarse approximation of the neural generators since such approach neglects the known modulations of posterior α activity through long-range and large-scale connectivity (Palva and Palva, 2007; Scheeringa et al., 2012). The neural generators of α activity have a long history (Adrian and Matthews, 1934): whether α generators are intrinsically cortical or driven by the thalamus is a debate initiated in the mid-twentieth century (Bremer, 1949; Kristiansen and Courtois, 1949). This question is still ongoing as both thalamocortical and intracortical generators of α activity appear to coexist. Corticothalamic α is well documented (Feige et al., 2005; Lőrincz et al., 2009; Vijayan and Kopell, 2012) and so is α activity in deep cortical layers (Buschman and Miller, 2007; Wang, 2010; Buffalo et al., 2011). The propagation of α can also be corticothalamic (Halgren et al., 2019). With the current experimental design, we did not investigate the generative sources of α activity and future experiments will target a better understanding of neural sources contributing to the observed episodic timing effect, including the possible relation between α burst activity and α traveling waves (Halgren et al., 2019; Davis et al., 2021; Das et al., 2022). Nevertheless, it is likely that the α bursts characterized here reflect participants’ internal stream of thoughts during quiet wakefulness (Sadaghiani and Kleinschmidt, 2016; Halgren et al., 2019) or mind-wandering (Compton et al., 2019) and be elicited by as part of larger functional networks (Raichle, 2015; Sadaghiani and Kleinschmidt, 2016; Buckner and DiNicola, 2019).

### Absence of replication after task engagement: time-on-task effects and contextual changes

We replicated the relation between spontaneous α burst time and retrospective time estimate at the beginning (Exp. 1) but not at the end (Exp. 2) of an experimental session. The failure to replicate was not an outcome of trivial factors, which we thoroughly evaluated. For instance, the sample size exceeded the threshold recommended by our sensitivity analysis in the first experiment (Extended Data Fig. 2-4). Although we used two different EEG systems, their use was orthogonal to the conditions that were tested and, for instance, the data from half of participants tested in Exp. 2 were acquired with the same EEG settings as Exp. 1. Additionally, the localization of α burst activity in Exp. 2 was comparable with that observed in Exp. 1 (Fig. 2*B*) and in the original MEG study (Azizi et al., 2023). Thus, scalp-level α activity analyzed in Exp. 2 did not appear entirely different from the one analyzed in Exp. 1.

One major expected impact of testing participants at the end of an experimental session is mental fatigue, which has been associated with spectral changes in α but also θ activity (Boksem et al., 2005; Wascher et al., 2014; Tran et al., 2020). Time-on-task effects have also been reported in which α power increases, and α peak frequency decreases, in the course of an experimental task (Benwell et al., 2019). Seeing that the cerebral marker for episodic timing under investigation is α power, the increase of α power predicted by time-on-task should have led to an increase in retrospective time estimate or a possible disappearance of the linear relation between α and retrospective time estimation. As in the initial study (Azizi et al., 2023) and in Exp. 1, participants in Exp. 2 significantly underestimated the duration spent in resting state. This supports the notion that participants did not pay attention to time during the recording and that their behavioral trend was comparable with conditions before an experimental session. However, we did find partial evidence of time-on-tasks effects in EEG data with a significant increase in α power between the beginning and the end of a recording session. This α power increase predicted that participants would underestimate elapsed time less or perhaps overestimate it. This is not what we found. Interestingly, we did observe that the increase in α power was attributable to sensors that were not part of the original parietal α cluster and that were instead more occipital. Thus, one possible explanation for the nonreplication is that the neural generators contributing to the α recorded at the scalp may have changed in the course of the experimental recording. No anatomical MRI of participants was acquired, which prevented precise source reconstruction of the EEG data to disambiguate this aspect, but future work will address this.

One complementary explanation for the vanishing relation between α activity and retrospective time estimation at the end of an experimental session appeals to the contextual change hypothesis, which predicts that retrospective time estimation is influenced by the amount of contextual change experienced during the time interval (Block and Reed, 1978). Under this working hypothesis, the brain does not track time per se but instead reconstructs time based on the memory for changes or mental states that occurred during that lapse of time (Ornstein, 1969). Accordingly, duration estimates have often been reported to increase with the amount of contextual changes that have been experienced by participants (Block, 1978, 2014; Zakay and Block, 1997), and high-arousal and emotionally salient events can also increase event segmentation thereby leading to longer remembered durations (Zakay et al., 1994; Frederickx et al., 2013; Wang and Lapate, 2025). At the beginning of an experimental session, the sequence of events is rather standard (welcoming to the lab, ethical approval, setting-up, explanations of the procedure) but more variability is expected by the end of an experimental session. One possibility is that the experimental context affected the storage and/or the retrieval of temporal information during the subsequent resting state in a manner that we did not experimentally control for. The 90-min-long experiments were segmented into shorter blocks and breaks, eliciting temporal clues and landmarks that could have influenced subsequent participants’ estimates, and also their resting state dynamics. At the time participants received the question, contextual interference effects could impact the correlation between α episodic timing and retrospective duration estimate. Future studies will exploit this direction of research as the literature has shown that parametric changes during minute long experiences affect subsequent temporal memory (Lositsky et al., 2016; Montchal et al., 2019; Brunec et al., 2020). For instance, Brunec et al. (2020) showed that memory of duration increases with the number of turns that an individual took during spatial navigation. Similarly, Lositsky et al. (2016) showed that the more event boundaries during an interval, the longer participants will estimate it to be.

Additionally, tasks tested in Exp. 2 involved timing, either explicitly or implicitly, which may have resulted in orienting participants’ attention to time. However, we think it is very unlikely because participants underestimated the retrospective duration as would be expected when not paying attention to time (Polti et al., 2018; Nicolaï et al., 2024). Recent findings also showed that participants who mind-wandered tend to underestimate the duration of events (Terhune et al., 2017). Still, the implication of the timing system for such a long recording period (90 min) may have influenced the dynamics of brain activity to the extent that resting-state dynamics during the episodic timing block and the retrieval of stored information functionally dissociated. Although difficult to operationalize mechanistically, and perhaps unparsimonious, we cannot disregard this possibility, which requires empirical validation. This would require a particular experimental design in which the same participants are tested before and after taking part in timing tasks or in nontiming tasks.

A plausible explanation is that by the end of the experimental session, participants have developed temporal expectations for the duration of an experimental block. During the debrief of Exp. 2, 21.3% of participants effectively reported using the preceding experimental blocks or break times (Fig. 1) as a reference for their retrospective duration estimation. The temporal expectation response strategy would affect the decision stage so that the retrospective estimation of duration resulted from the comparison between stored duration memories of past blocks with the just elapsed one (Kaju and Maglio, 2022). This comparison strategy would account well for the observed behavioral outcomes: retrospective durations were underestimated not by lack of attention to time but due to a shorter than expected experimental block. This strategy would also suggest that some participants may have paid attention to elapsing time during the recording, in which case the relation between α burst time and retrospective duration is predicted to vanish (Azizi et al., 2023). Prior studies (Jones and Boltz, 1989; Tanaka and Yotsumoto, 2017; Jones, 2019) predict that the passage of time would feel faster for participants in Exp. 2 than in Exp. 1 or Exp. 3 due to the existence of priors on the length of an experimental block. This is indeed what we observed (Extended Data Fig. 2-3), suggesting that by the end of an experimental session, participants may have used such a comparison strategy. Implicit statistical learning and mind-wandering appear to be decoupled (Massar et al., 2020) so that the reported dissociation between α dynamics and retrospective duration observed here may result from the decisional aspect, not from the temporal dynamics of the mind-wandering (itself affected by time-on-task effects).

Last, adding on to time-on-task fatigue, context, and the response strategy, the preceding task demands during the recording session could also influence the quality of subsequent resting states and possibly alter the content of mind-wandering, which is another possibly exploitable line of research that we have not controlled for here.

Hence, for all practical purposes, it is important to better understand the context and the timing at which spontaneous α activity and retrospective time estimations are being recorded during an experimental setup. Retrospective timing tasks, as a single-trial and few-minutes long experiment, can easily be included in a series of experimental tasks (Chaumon et al., 2022). One clear recommendation at this stage would be to test retrospective timing tasks as early as possible in an experimental session to prevent fatigue, uncontrolled contextual effects on episodic timing or mind-wandering.

### Resting in a VR environment does not replicate

In Exp. 3, we wanted to test the generalizability of the episodic timing marker in VR and the possibility that the size of VR would impact time estimation and the marker. Neither the replication nor the effect of VR size was found on retrospective duration or on its relation to α activity.

VR is being increasingly used in timing research (Tobin and Grondin, 2009; Tobin et al., 2010; Riemer et al., 2014; Jording et al., 2022; Lamprou-Kokolaki et al., 2024). One legitimate question is whether VR is comparable with real-life experiments (Bogon et al., 2024), an issue that has also been raised in spatial cognition (Taube et al., 2013). When estimating time intervals in the order of seconds, time in VR appears generally similar to real life (Bogon et al., 2024) but, interestingly, VR seems to alter the expectations about the duration of physical processes: for instance, how long a bottle takes to empty will differ in VR and in real-life, but not abstract time perception (Bogon et al., 2024). The realism of the VR environment can also affect time estimates, sometimes making them less accurate than in real life (Mioni and Pazzaglia, 2023; Bogon et al., 2024). Yet, emotional arousal and content impact subjective time perception suggesting that one's experience of VR, rather than the environment itself, is what affects time perception (van der Ham et al., 2019; Mioni and Pazzaglia, 2023). This may imply large variability in how individuals relate to time in VR. Rutrecht et al. (2021) showed that in VR, the flow state or loss of the sense of time” (Csikszentmihalyi, 2013) correlated with reduced time awareness and faster subjective time passage but did not correlate with the accuracy in retrospective duration estimation (Rutrecht et al., 2021). This corroborates that the subjective experience of the felt speed of the passage of time differs from the ability to accurately judge elapsed duration (Wearden, 2015; Droit-Volet et al., 2017; Lamprou-Kokolaki et al., 2024). As seen previously, if the two phenomenological outcomes and measures are distinct, their underlying mechanisms may not be fully unrelated.

The first major difference between Exp. 3 and other experiments is that participants did not significantly underestimate the duration spent in VR, whether in the small or in the large VR room. The lack of time underestimation in VR indicates that participants may have prospectively timed. Unlike in the other two experiments, participants were seated in a room where a lamp was placed in front of them. As the start of the experiment was indicated by the lighting of the lamp, this stimulation may have yielded prospective (implicit or explicit) timing and temporal expectations as to when the lamp would next be lit up. Alternatively, participants may also have experienced boredom, known to lengthen time estimation but also slow the felt passage of time (Watt, 1991; Droit-Volet and Meck, 2007), which is not what we found. Second, the size of the VR did not significantly impact the retrospective time estimation as would have been expected through other studies (DeLong, 1981; Riemer et al., 2014). Two differences can explain this incongruence: our study tested a different time scale and used a retrospective, not a prospective paradigm. Additionally, temporal distortions previously reported to be affected by the scale of the environment were realized in nonstatic environments. Hence, behavioral outcomes already predicted a possible lack of replication, seeing that an individual's experience in a VR environment may already alter temporal phenomenology. Prospective timing was predicted to alleviate the relation between α and temporal estimation (Azizi et al., 2023).

It is perhaps noteworthy mentioning that the quality of EEG recordings while in VR was comparable with the other experiments, including the global statistics of α and relative α burst times. The topographic maps of α, θ, and β frequency bands during quiet wakefulness were also consistent across experiments. Thus, the absence of relation between α bursts and retrospective time estimation is unlikely to be attributed to differences in signal quality.

### Conclusions

In three EEG experiments, we aimed to extend previous work demonstrating the involvement of oscillatory α bursts in episodic timing. We replicated the underestimation of the retrospective duration estimation of a resting state and the positive linear relation between retrospective duration estimates and relative α burst time. However, we failed to find this relation when participants were tested at the end of an experimental session due to the contextual shaping of temporal expectations yielding different cognitive strategies in the estimation of retrospective duration. The relation did not generalize to VR, as participants may have likely oriented their attention to time in this environment. Overall, additional work is needed to assess the robustness of the link between spontaneous α activity and the tracking of episodic timing.

## Synthesis

Reviewing Editor: Anne Keitel, University of Dundee Decisions are customarily a result of the Reviewing Editor and the peer reviewers coming together and discussing their recommendations until a consensus is reached. When revisions are invited, a fact-based synthesis statement explaining their decision and outlining what is needed to prepare a revision will be listed below. The following reviewer(s) agreed to reveal their identity: Andreas Wutz, Audrey Morrow.

The manuscript has been reviewed by two expert reviewers and the editor. Both reviewers agreed that the study is a valuable contribution to the field. Both reviewers also had a major and several minor comments. You will find a summary of both reviewers' comments below. Please respond to each comment in a point-by-point manner.

Overall assessment for your information

(1) This study uses EEG to (partly) replicate and extend previous MEG work showing that the relative time spent in an alpha-frequency burst state correlates with retrospective duration judgments. The authors conducted three experiments, in which the participants' eyes-open resting state activity was recorded. Afterwards, the participants were asked to estimate the duration of the resting state recording. In experiment 1, they replicate the finding that subjective time is experienced as shorter when the resting state recording contained less alpha burst cycles. Experiment 2 did not replicate this effect, which is explained by contextual factors, namely that the participants underwent timing tasks before the resting state recording and thus were biased towards prospective timing. In Experiment 3, the original findings were not replicated in a virtual reality environment. In sum, the authors discuss their heterogeneous results with respect to the importance of experimental context for establishing robust relationships between alpha activity and temporal experiences.

This is a fine study replicating previous MEG work by means of EEG. Because the authors use largely the identical methodology to the previous work, there is not much to criticize in terms of the employed methods, analysis and statistics (but please see specific comment about the use of cluster-based permutation tests below). The reported findings are clear and are adequately discussed. The figure design is very nice and overall the paper is well written. Of course, it is always hard to explain partial replications and null effects in one paper, such that the stated reasons sometimes come across as post-hoc. Nevertheless, it makes much sense to me that contextual factors play a key role both for alpha and subjective time judgments. I have a couple of mainly minor points, alongside with two more major comments. One concerns the proper use of cluster-based permutation approaches, and the other makes a suggestion for additional analyses. As you can read below, this suggestion naturally follows from the described theoretical background about the relationship of alpha burst states and experienced time.

(2) This manuscript not only replicates prior work showing the relationship between alpha bursts and episodic timing, but also explores this relationship in two novel task paradigms. The paper explores the neural dynamics underlying retrospective duration estimates in a number of contexts, replicating prior work and evaluating novel task paradigms that may affect duration estimates. The initial experiment replicated a prior finding that alpha bursts were associated with retrospective duration estimates following quiet wakefulness. However, these associations were not present following a timing task nor a VR context, suggesting the attentional allocation to a task or environment may have affected the cognitive or neural mechanisms underlying episodic timing. The authors thoroughly outline their methodologies and analyses, and took precautions to ensure consistency in data collection and measures. The authors also do well explaining the results, implications, and future directions, and present an overall strong paper. I believe there could be some improvements around how Experiment 2 is presented throughout the paper, as it was not clear to me the different tasks or experimental setups until the end of the analysis / beginning of results. However, my only other comments are very minor suggestions related to clarity or wording, listed below.

Major comments - please respond to these

1. It makes much sense to relate the present work to the contextual change hypothesis by Block (1985), which states that the more context changes, the longer time is felt. Moreover, it is also claimed that alpha bursts reflect discrete brain and mental states, "which could serve as neural markers of such internal contextual changes during quiet wakefulness" (p. 3)

This leads to a straightforward hypothesis, which is not yet investigated in the paper. It suggests that the more transient alpha bursts are detected in the recording, and thus the more changes from one state to another, the longer time should be experienced. This is different from the reported findings, because it captures the number of changes between burst and non-burst states rather than the overall time spent in a burst state. We would very much appreciate, if this additional analysis were performed, because we are very curious about its outcome. Nevertheless, we do want to acknowledge that this is a suggestion, whose outcome does not alter the validity of the reported work and thus it will not influence the overall assessment.

2. The use of the cluster-based permutation procedure is questionable for the reported purpose. It is not straightforward to use it for testing differences against a fixed, parametric value, as done here by contrasting activity across all EEG electrodes against the (one) average over all EEG electrodes. This is so, because the permutation test is based on the null hypothesis of exchangeability between experimental conditions, which is not met if you randomly permute between data and one single data point (aka for a one-sample test).

Please see here for an in-depth discussion:

https://mailman.science.ru.nl/pipermail/fieldtrip/2018-August/025180.html

Nevertheless, we also want to make clear that the authors use the cluster-based permutation approach here as a localizer method and not for statistical inference. Moreover, we are also convinced that, based on the shown topographies, the reported findings of stronger alpha activity and more alpha bursts over occipital-parietal sensors is legit, rendering those comments less concerning. Technically, however, the authors' approach is not legit.

3. Both reviewers found it difficult to understand the flow of events, especially for Experiment 2. Specifically, there could be more clarification around the structure of Expt. 2, given the different tasks and the subsetting of participants for resting-state recordings. It wasn't very clear how/when participants were assigned to the different tasks, or the tasks themselves. Clarifying this in the Experimental Design section makes the most sense, but as the Participant section came first, this is where questions about the design came up.

For example, in the Participants section, the authors state, "Among these 45 participants, 21 were recorded during a resting-state alternating eyes-closed and eyes-open before taking part in an explicit timing task. This subset of data was analyzed to compare the α burst before and after an explicit timing task," which seems to be a separate subsetting than the task. Not until the Results is it stated, "Participants in Exp. 2 either performed a temporal adaptation task (N = 25) or an implicit timing task (N = 23) before the resting-state constituting the retrospective duration to be estimated." It is challenging to follow the design when the details are spread out like this, and the paper would benefit from adding a section with clear and concise outlining of Expt. 2 and referencing to it in the Participants section.

Minor comments - please respond to these

1. The number of participants mentioned in the abstract (N=147) does not match with the actually used participant pool (N=132). This is misleading.

2. I find the following statement odd without further explanation (p. 6):

"The retrospective time task consisted of an eye-open resting-state EEG recording followed by a question and a questionnaire."

Which questionnaire was used? And, isn't a questionnaire a collection of many questions?

3. I fail to understand the following statement (p. 9):

"To prevent a high number of small data cropping and edge artifacts in burst detection, the minimum length of a good data span was set to 500 ms, and so was the minimum length of a bad data span."

4. What do you mean by "integrated power" (p. 10 and other). Is it the average over time and frequencies?

5. I don't agree with the following statement (p. 12):

"As a possible interpretation of this variable, the more bursty the signal is, the more stationary the signal is in the time domain. On the contrary, a 0% bursting signal means that no α activity is detected."

Isn't a 0% burst signal also "stationary" in a non-burst state?

6. Please streamline your use of the Wilcoxon signed-rank (aka a dependent samples test) vs. the Wilcoxon rank-sum (aka an independent samples test, Mann-Whitney U test). At least in the Figure 1 legends, you have mixed them up.

7. Further about Figure 1 legends: The p-values in the legend do not match with the p-values reported in the text. Moreover, there is no "red dotted line" in Figure 1D.

8. Please specify the null model for the likelihood ratio test.

9. About Figure 3 legends: You mention "z-scored power" but I cannot find any description of z-scoring in the methods.

10. The penultimate line of abstract reads a little awkwardly, and is perhaps missing a conjunction? E.g. should be "Overall, while EEG captures the..." or "Overall, although..."

11. In the significance statement, not clear on what is meant by, "Virtual reality environments also affected behavior in a way that suggested prospective timing which the marker is known not to capture." Did their behavior suggest they were engaging in prospective timing? It seems authors expand on which marker/why it is known to capture prospective timing in the discussion, but it would be helpful to include a little more detail here.

12. "This is important as this episodic timing marker can serve AS A clinical diagnosis seeing that time perception is altered in numerous pathologies (Hinault et al., 2023)". (add as a)

13. In participants, age data is unclear as it is currently written - is age the mean age? Does the +/- indicate the full age range or SD?

14. Why were only 21 included in the explicit timing task? This sample size doesn't meet the power analysis requirement, so it would be good to justify. Also, how many were in the explicit timing task and how many in the implicit task? This is stated in the Results, but would be helpful to have in Participants where you are describing all the other sample sizes.

15. Beginning of Experimental design, should read: eyes-open resting-state EEG

16. How many points on the felt-speed Likert?

17. PoTJ acronym is defined in a caption but not in the body of the paper

18. "Contrary to Exp. 1 and Exp. 2, we found three outliers in the distribution of duration estimates IN EXP. 3 (see Methods)." (specify where outliers found)

19. "THERE WERE no significant underestimations of elapsed duration in Exp. 3, when participants performed the resting-state recording in either virtual rooms." (add there were)

## Author Response

Major comments 1. It makes much sense to relate the present work to the contextual change hypothesis by Block (1985), which states that the more context changes, the longer time is felt. Moreover, it is also claimed that alpha bursts reflect discrete brain and mental states, "which could serve as neural markers of such internal contextual changes during quiet wakefulness" (p. 3) This leads to a straightforward hypothesis, which is not yet investigated in the paper. It suggests that the more transient alpha bursts are detected in the recording, and thus the more changes from one state to another, the longer time should be experienced. This is different from the reported findings, because it captures the number of changes between burst and non-burst states rather than the overall time spent in a burst state. We would very much appreciate, if this additional analysis were performed, because we are very curious about its outcome. Nevertheless, we do want to acknowledge that this is a suggestion, whose outcome does not alter the validity of the reported work and thus it will not influence the overall assessment.

We thank the Reviewers and the Editor for raising this important point. There are several ways to understand the proposed analysis.

A first one would be to compare bursting activity intra-individually, that is comparing whether the variation in alpha burst rate in an individual's data predicts shorter or longer retrospective time estimations. This analysis is not currently possible seeing our approach is inter-individual.

A second one, which we believe is what is proposed, is to take the same inter-individual approach to characterize the number of bursts, rather than the relative duration of bursting activity as currently reported. This specific analysis was performed in the original paper (Azizi et al., 2023), where the number of bursts was quantified (thus, directly testing the contextual change hypothesis). The alpha power, the burst amplitude, and the relative burst time or combination thereof were also tested (Azizi et al., 2023; Fig. 3, Table 1). The authors reported that the best predictor of the retrospective time estimates (rTE) was the relative burst time. The authors also tested the number of bursts and the relative burst time remained the best predictor (unpublished; personal communication).

As requested, in the current Exp.1, we followed a similar analysis as in Azizi et al., 2023 and now report the outcomes in Extended Data Figure 2-5, and Table 2-1 and referred to in Figure 2.

We tested alpha power, burst amplitude, and the number of bursts (reported as bursts per second to obtain a relative measure; thereafter "burst rate"), which are all collinear measurements. Specifically, the relative burst time and the burst rate were highly correlated (⍴(24) = 0.70, p < 0.001), as expected. We also found a significant correlation between the burst rate and rTE (Extended Data Figure 2-5) as would be predicted.

However, while the general outcomes are comparable to those of the original study, in that the relative burst time was a better predictor of the rTE than all other covariates, the implication of the burst rate is more difficult to interpret from the analyses. Two distinct measures of prediction quality (⍴, AIC) yield incongruent outcomes in that the Spearman's correlation (⍴) would say that the relative burst time is a better predictor, whereas the AIC would lean towards the burst rate being a better model. Specifically, while Spearman's correlation with the rTE was higher for the relative burst time (⍴(24) = 0.54, p = 0.005; see Fig. 2) than for the burst rate (⍴(24) = 0.45, p = 0.022; see Extended Data Fig. 2-5), the AIC was lower for the linear model with the burst rate as rTE predictor (Table 2-1: AIC = -3.79 and AIC = -3.95, respectively). Thus, in the current EEG study, there is an ambiguity regarding which predictor is better.

Despite this ambiguity, we believe that the relative burst time remains the more robust effect for two main reasons: one from the signal modelisation perspective, and the other from a statistical perspective.

With respect to the modelisation of the bursts, the cycle-by-cycle method was tuned for "overall time spent in a burst state" rather than for the individual segmentation of bursts, i.e., for the relative burst time and for burst rate. This is because we noticed that the cycle-by-cycle method can split a burst due to cycle characteristics. To overcome this issue, we established specific criteria for cycle inclusion (see the "Cycle-by-cycle analysis and burst detection" paragraph in our Methods section). Accordingly, the correlation between alpha power and relative burst time (⍴(24) = 0.97, p < 0.001) is higher than the correlation between alpha power and burst rate (⍴(24) = 0.63, p < 0.001). Hence, we believe that the relative burst time is a more robust measurement of the temporal dynamics of alpha activity than the burst rate or number of bursts per second.

From the statistical perspective, Spearman's correlation is a non-parametric measure that is less sensitive to outliers and deviations from normality or linearity. A Shapiro-Wilk test showed that, while the relative burst time distribution did not deviate from normality (W = 0.97, p-value = 0.60), the burst rate distribution did (W = 0.92, p-value = 0.040). Importantly, residuals of all linear models were normally distributed (Shapiro-Wilk test, p > 0.2), validating the use of the AIC for model comparisons. From that perspective, the Spearman's test appears more reliable and favors the relative burst time interpretation.

All-in-all, we acknowledge the relevance and importance of testing the burst rate with regard to the contextual change hypothesis, but our analyses and results rather support the overall time spent in a burst state as the main predictor of retrospective time estimates.

We are unsure whether this additional analysis should be left in the MS as Extended Data. We leave it to the discretion of the Editor to decide whether or not we should keep it. In the meantime, we made sure that it was mentioned in the main text (Figure 2).

2. The use of the cluster-based permutation procedure is questionable for the reported purpose. It is not straightforward to use it for testing differences against a fixed, parametric value, as done here by contrasting activity across all EEG electrodes against the (one) average over all EEG electrodes. This is so, because the permutation test is based on the null hypothesis of exchangeability between experimental conditions, which is not met if you randomly permute between data and one single data point (aka for a one-sample test).

Please see here for an in-depth discussion: https://mailman.science.ru.nl/pipermail/fieldtrip/2018-August/025180.html Nevertheless, we also want to make clear that the authors use the cluster-based permutation approach here as a localizer method and not for statistical inference. Moreover, we are also convinced that, based on the shown topographies, the reported findings of stronger alpha activity and more alpha bursts over occipital-parietal sensors is legit, rendering those comments less concerning. Technically, however, the authors' approach is not legit.

We thank the Editor and the Reviewers for bringing this issue to our attention. We agree that the cluster-based permutation is not the ideal approach for channel selection but it was efficient in the original paper, in which it was used as an alternative to simple thresholding. We could have used a bootstrapping approach to inform the spatial selectivity of the alpha activity, but the SNR on such short EEG epoch data would likely not have been as reliable. As readily acknowledged by the Editor and the Reviewers, this approach was used solely to reduce the dimensionality of the data and capture the alpha-sensitive sensors. To acknowledge this, we have added a sentence in the Methods section stressing that no inference was made on this analysis: "Importantly, this procedure was not used to make inferences, as we tested against a fixed value, but only to select a subset of channels." (p.11) 3. Both reviewers found it difficult to understand the flow of events, especially for Experiment 2. Specifically, there could be more clarification around the structure of Expt. 2, given the different tasks and the subsetting of participants for resting-state recordings. It wasn't very clear how/when participants were assigned to the different tasks, or the tasks themselves. Clarifying this in the Experimental Design section makes the most sense, but as the Participant section came first, this is where questions about the design came up.

For example, in the Participants section, the authors state, "Among these 45 participants, 21 were recorded during a resting-state alternating eyes-closed and eyes-open before taking part in an explicit timing task. This subset of data was analyzed to compare the α burst before and after an explicit timing task," which seems to be a separate subsetting than the task. Not until the Results is it stated, "Participants in Exp. 2 either performed a temporal adaptation task (N = 25) or an implicit timing task (N = 23) before the resting-state constituting the retrospective duration to be estimated." It is challenging to follow the design when the details are spread out like this, and the paper would benefit from adding a section with clear and concise outlining of Expt. 2 and referencing to it in the Participants section.

We thank the Reviewers for helping us clarify the Methods. We have now included a new Figure (Figure 1) to better explain the configuration of the different experimental protocols. References to this Figure have been added in both the Participant and the Experimental Design sections.

Minor comments 1. The number of participants mentioned in the abstract (N=147) does not match with the actually used participant pool (N=132). This is misleading.

This has now been clarified in text. 147 participants were recruited, and 128, not 132 as previously indicated, participants were included in the final analyses (sample sizes in each experiment were correctly reported). The abstract and the main text have been corrected to reflect the sample size used, not the number of recruited participants.

2. I find the following statement odd without further explanation (p. 6): "The retrospective time task consisted of an eye-open resting-state EEG recording followed by a question and a questionnaire." Which questionnaire was used? And, isn't a questionnaire a collection of many questions? The sentence has now been clarified and replaced with, "The retrospective time task consisted of an eyes-open resting-state EEG recording followed by a questionnaire as detailed in the next paragraph." 3. I fail to understand the following statement (p. 9): "To prevent a high number of small data cropping and edge artifacts in burst detection, the minimum length of a good data span was set to 500 ms, and so was the minimum length of a bad data span." This statement has now been clarified p. 9: "To prevent fragmentation and edge artifacts of burst detections, we set the "min_length_good" parameter in mne.preprocessing.annotate_muscle_zscore to .5. This means that consecutive muscle artifacts separated by less than 500 ms were merged together, and artifacts lasting less than 500 ms were discarded." 4. What do you mean by "integrated power" (p. 10 and other). Is it the average over time and frequencies? This is correct. Following the binning over frequencies in data analysis, the integrated power is the average power over frequency in the alpha frequency range. To avoid any confusion, all mentions of "integrated power" have now been replaced with "averaged power".

5. I don't agree with the following statement (p. 12): "As a possible interpretation of this variable, the more bursty the signal is, the more stationary the signal is in the time domain. On the contrary, a 0% bursting signal means that no α activity is detected." Isn't a 0% burst signal also "stationary" in a non-burst state? We agree that this statement was a little extreme in its view. We have now replaced it with the following statement: "As a possible interpretation of this variable, the more bursty the signal is, the more rhythmic or sustained the signal is in the time domain. On the contrary, a 0% bursting signal means that no α activity is detected." 6. Please streamline your use of the Wilcoxon signed-rank (aka a dependent samples test) vs. the Wilcoxon rank-sum (aka an independent samples test, Mann-Whitney U test). At least in the Figure 1 legends, you have mixed them up.

We thank the Reviewers for catching the mixing. All mentions of the Wilcoxon signed-rank and rank-sum tests have now been carefully checked and corrected when necessary.

7. Further about Figure 1 legends: The p-values in the legend do not match with the p-values reported in the text. Moreover, there is no "red dotted line" in Figure 1D.

The legends of Figure 1 have been replaced by the corrected p-values (which were the ones reported in text). The "red dotted line" phrase has been corrected and changed to "grey dotted line." Additionally, we replaced panel E Figure 1 with its corrected version. In particular, the correlation coefficient should read "⍴ = 0.54" instead of "⍴ = 0.53", as it was already reported in text (actual value with 4 digits: ⍴ = 0.5376).

8. Please specify the null model for the likelihood ratio test.

The likelihood ratio test, used for the ordinal regression, has now been clarified in the Methods section (p. 14) and defined as: "the null model, which included only the intercept." In the results section, we provide further details on the model outcomes (p. 17).

9. About Figure 3 legends: You mention "z-scored power" but I cannot find any description of z-scoring in the methods.

We apologize for the confusion. No z-scoring was used in the analyses reported in Figure 3. The Figure legend has been corrected accordingly and now reads as " Localization of θ band activity using a permutation t-test on the averaged power in Exp. 1." 10. The penultimate line of abstract reads a little awkwardly, and is perhaps missing a conjunction? E.g. should be "Overall, while EEG captures the..." or "Overall, although..." A conjunction has been added to this sentence: "Overall, while EEG captures..." 11. In the significance statement, not clear on what is meant by, "Virtual reality environments also affected behavior in a way that suggested prospective timing which the marker is known not to capture." Did their behavior suggest they were engaging in prospective timing? It seems authors expand on which marker/why it is known to capture prospective timing in the discussion, but it would be helpful to include a little more detail here.

We thank the Reviewers and the Editor for this helpful comment. We have now replaced the sentence with "Virtual reality environments may have fostered prospective timing behavior, which the alpha marker is known to be insensitive to." 12. "This is important as this episodic timing marker can serve AS A clinical diagnosis seeing that time perception is altered in numerous pathologies (Hinault et al., 2023)". (add as a) Thank you, this sentence has been corrected.

13. In participants, age data is unclear as it is currently written - is age the mean age? Does the +/- indicate the full age range or SD? This has been corrected. The standard deviation is reported for age data and is now clearly stated, as (age = XX years old, SD = XX years), for all occurrences.

14. Why were only 21 included in the explicit timing task? This sample size doesn't meet the power analysis requirement, so it would be good to justify. Also, how many were in the explicit timing task and how many in the implicit task? This is stated in the Results, but would be helpful to have in Participants where you are describing all the other sample sizes.

In two places (p. 5 and p. 16), we mistakenly reported the sample size of Exp. 2.

We have now corrected, and we confirm that in Exp. 2, N = 23 participants were included in the explicit timing task, and N = 22 participants were included in the implicit timing task. We apologize for the confusion in the original MS. The added Figure in the Participant and Experimental Design sections (see major comment 3) now details the correct sample size for each task.

We agree that the sample size does not meet the initial power analysis requirement. However, the sample sizes are still above the minimum sample size defined by the sensitivity analysis (Extended Data Figure 2-4). More importantly, as outlined p.20 and p.21, the samples from the explicit and the implicit timing tasks were merged together and not analyzed separately (after ensuring homogeneity of the distributions). The sample size for the reported analyses is thus effectively N = 45 in Exp. 2.

15. Beginning of Experimental design, should read: eyes-open resting-state EEG This has been corrected in text.

16. How many points on the felt-speed Likert? A sentence has been added in the Experimental design to define the PoTJ and clarify the Likert scale (also, see comment 17): "They were asked to report the strategy they used to make their duration estimation and to judge their felt speed of the passage of time (PoTJ) on a 5-point Likert scale ranging from 1 (Very Slow) to 5 (Very Fast)." 17. PoTJ acronym is defined in a caption but not in the body of the paper A sentence has been added in the Experimental design to define the PoTJ and clarify the Likert scale (see comment 16).

18. "Contrary to Exp. 1 and Exp. 2, we found three outliers in the distribution of duration estimates IN EXP. 3 (see Methods)." (specify where outliers found) The sentence has been clarified as suggested. It now reads as: "Contrary to Exp. 1 and Exp. 2, we found three outliers in the distribution of duration estimates in Exp. 3. All outliers overestimated durations (see Methods)." 19. "THERE WERE no significant underestimations of elapsed duration in Exp. 3, when participants performed the resting-state recording in either virtual rooms." (add there were) Thank you, this sentence has been corrected.
