# Supplementary figures and images for: Spontaneous Oscillatory Activity in Episodic Timing: An EEG Replication Study and Its Limitations
Source: eNeuro. 2026 Jan 20;13(1):ENEURO.0332-25.2025. doi: 10.1523/ENEURO.0332-25.2025 (PMC12834324; doi:10.1523/ENEURO.0332-25.2025)

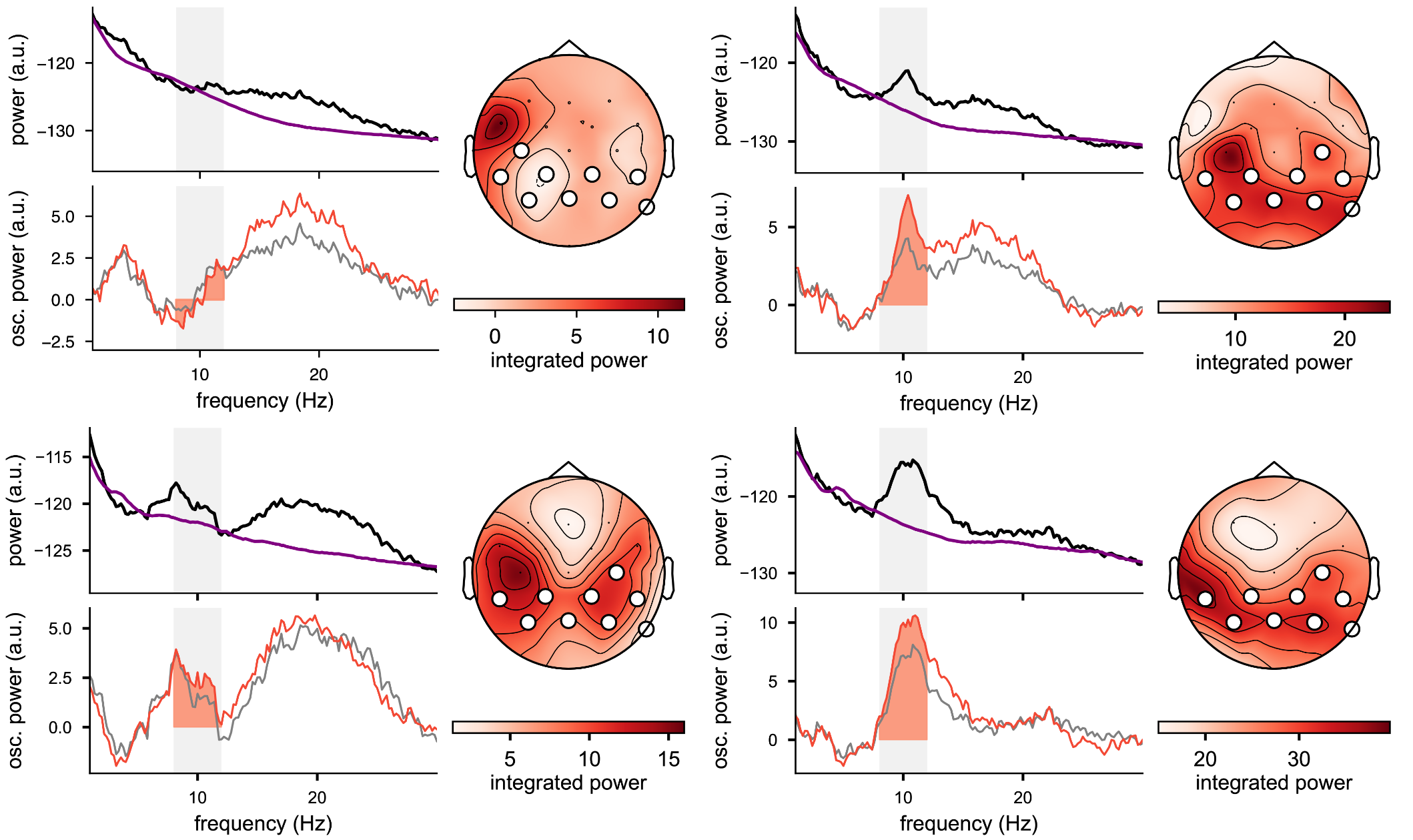

Supplement: Figure 2-1 — Topographic maps of excluded participants due to the lack of α activity in the cluster of channels localized by the cluster group. Download Figure 2-1, TIF file. [file eneuro-13-ENEURO.0332-25.2025-s001.tif]

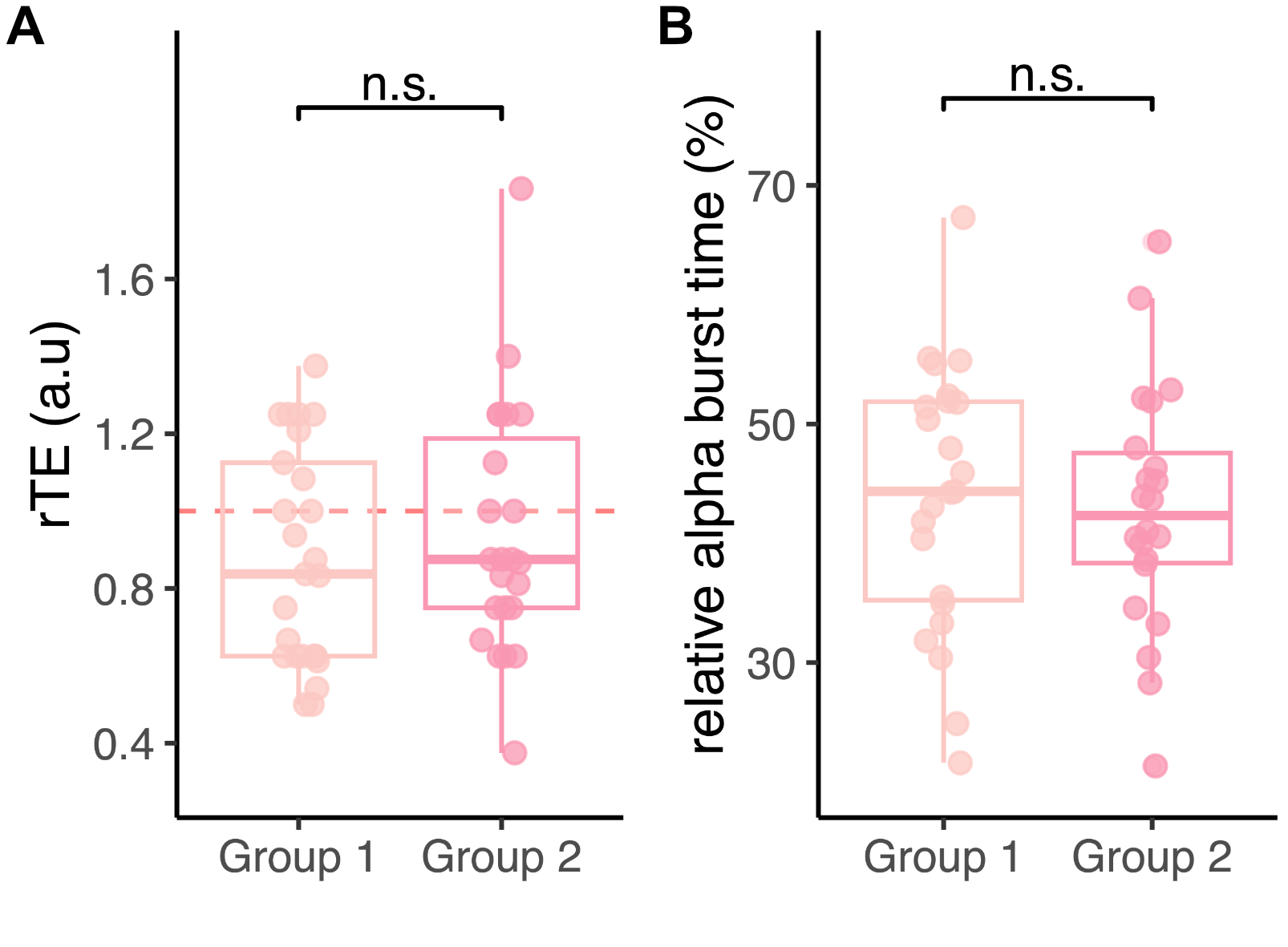

Supplement: Figure 2-2 — Exp. 2 data result from merging two homogeneous groups performing a resting-state EEG recording after a 90-minute timing task. (A) Behavioral distribution of Group 1 (after a temporal adaptation task) and Group 2 (after an implicit task). n.s. indicates a non-significant Wilcoxon rank-sum test (W = 226.00, p = 0.546) (B) Bursts distribution of Group 1 (after a temporal adaptation task) and Group 2 (after an implicit task). ns indicates a non-significant Wilcoxon rank-sum test (W = 278.00, p = 0.581). Download Figure 2-2, TIF file. [file eneuro-13-ENEURO.0332-25.2025-s002.tif]

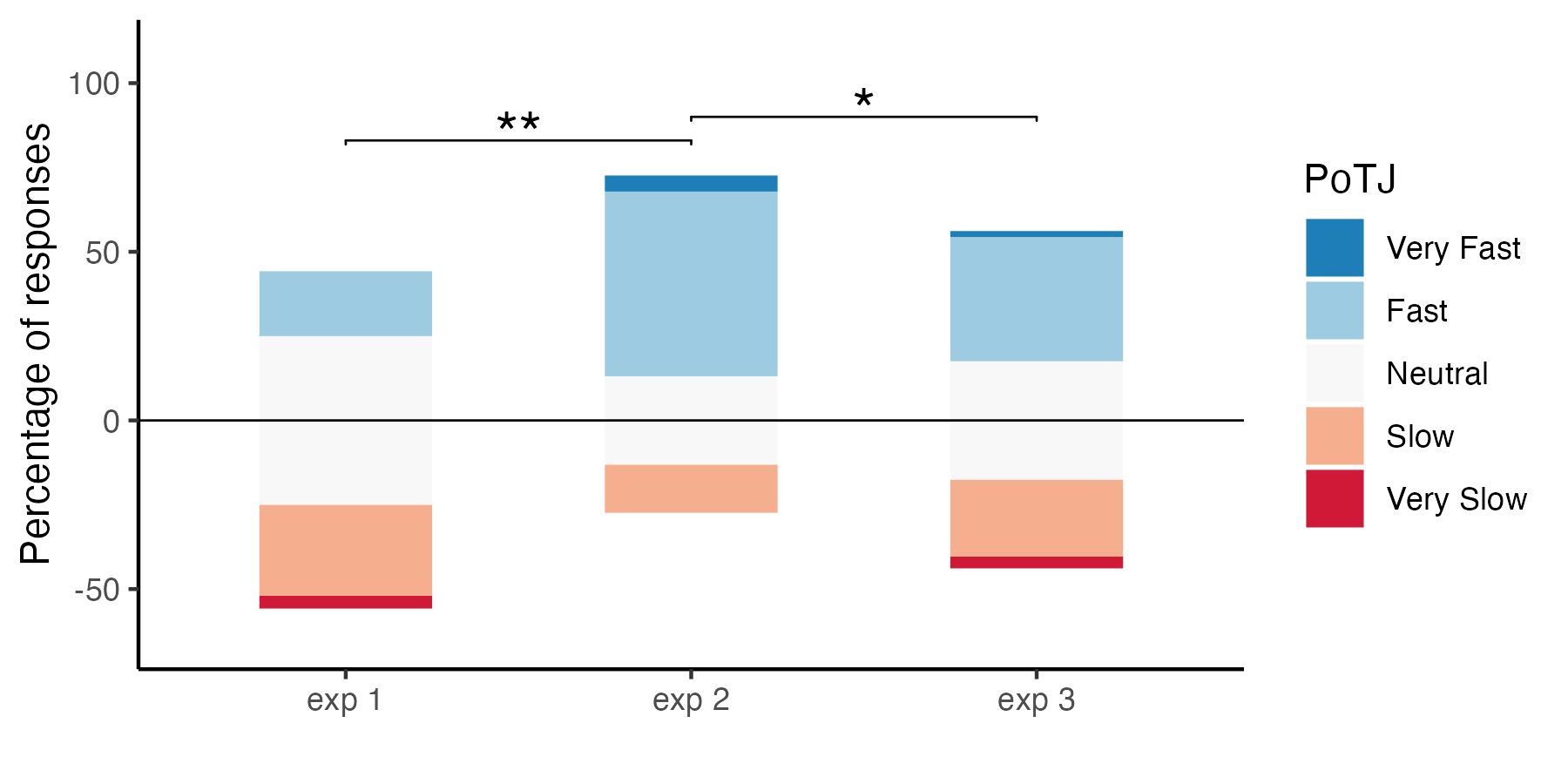

Supplement: Figure 2-3 — Felt passage of time judgments (PoTJ) were significantly faster in Exp. 2 than in Exp. 1 and in Exp. 3. Participants judged the experienced duration of the episodic time block on a 5-point Likert scale ranging from 1 (Very Slow, dark red) to 5 (Very Fast, dark blue). An ordinal logistic regression was computed with the PoTJ as the dependent variable and the experiment as the predictor. Significance was assessed through a likelihood ratio test against the null model (**p = 0.006; *p = 0.047). Download Figure 2-3, TIF file. [file eneuro-13-ENEURO.0332-25.2025-s003.tif]

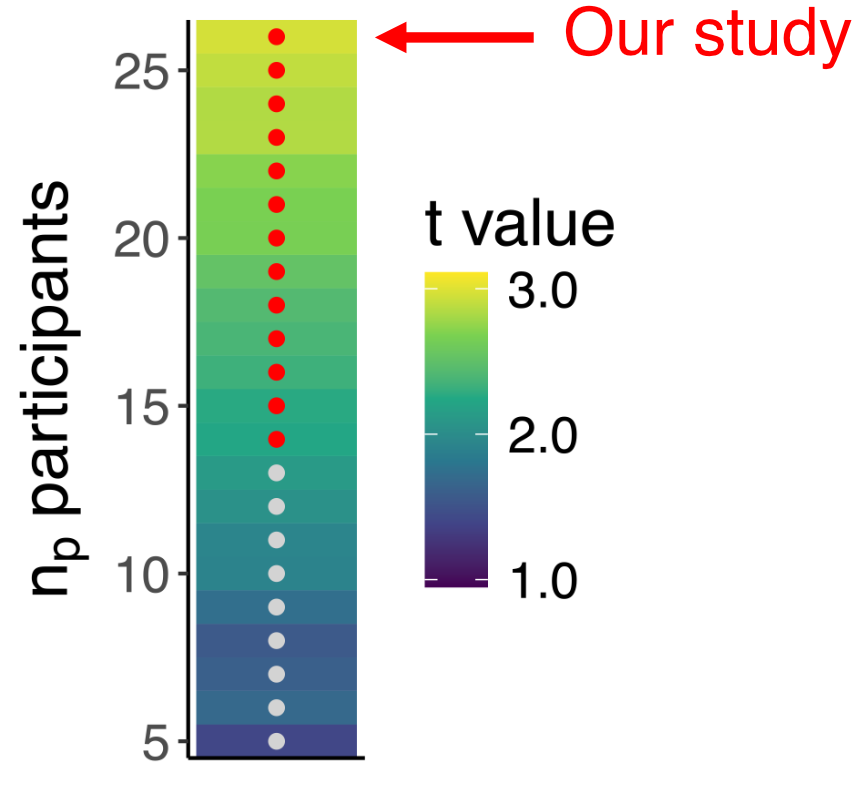

Supplement: Figure 2-4 — Sensitivity analysis of the relation between the retrospective time estimates (rTE) and the α bursts. Averaged t-value of the slope coefficient of the linear model for 100 repetitions of a sampling of np participants. np ranged from 5 to 26 (y-axis). Red dots indicate averaged t-values (over 100 repetitions) greater than the 97.5% quantile of the t distribution (with degrees of freedom of np − 2). Download Figure 2-4, TIF file. [file eneuro-13-ENEURO.0332-25.2025-s004.tif]

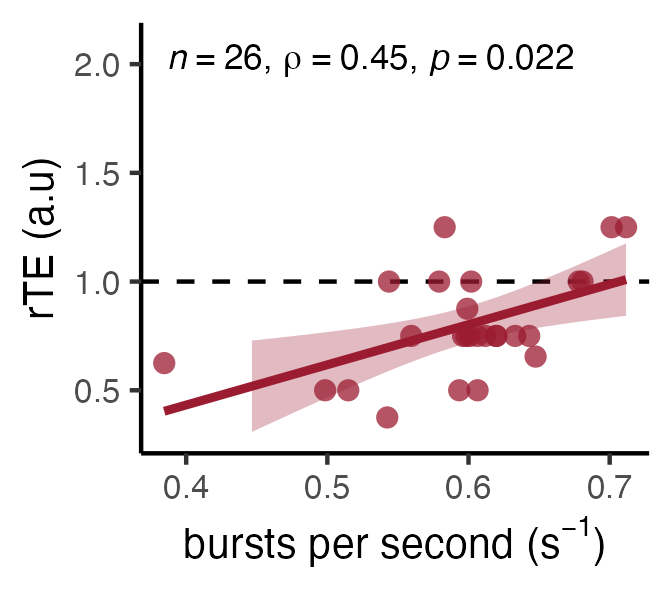

Supplement: Figure 2-5 — Spearman’s correlation between the number of α bursts per second and the rTEs in Exp. 1. The straight line is the regression line, and the shaded area is the 95% CI. Download Figure 2-5, TIF file. [file eneuro-13-ENEURO.0332-25.2025-s005.tif]
