# Supplementary material for: Spontaneous Oscillatory Activity in Episodic Timing: An EEG Replication Study and Its Limitations
Source: eNeuro. 2026 Jan 20;13(1):ENEURO.0332-25.2025. doi: 10.1523/ENEURO.0332-25.2025 (PMC12834324; doi:10.1523/ENEURO.0332-25.2025)
Supplement: Figure 2-6 — Model comparisons for predicting the rTE in Exp. 1 (N = 26). The ⍴ denotes Spearman's correlation coefficient. The F-statistic assesses whether the regression model to predict the rTE is better than a constant value, along with the corresponding p-value of the F-test. R2 represents the coefficient of determination, and lower values of the Akaike Information Criterion (AIC) indicate a better fit. Bold values indicate the best models, as determined by Spearman’s correlation and AIC. Note that the discrepancy of the best predictors according to Spearman’s correlation (non-parametric measures) and AIC (based on parametric models) can be explained by the differing robustness of the predictors: the relative burst time is more robust, whereas the number of bursts per second is more prone to segmentation artefacts (e.g., splitting a burst) depending on the threshold values described in the Methods section. Download Figure 2-6, DOCX file. [file eneuro-13-ENEURO.0332-25.2025-s006.docx]

| Predictor | ⍴ | F statistic | p-value | R^2^ | AIC |
| --- | --- | --- | --- | --- | --- |
| relative burst time | **0.54** | 8.86 | 0.007 | 0.27 | -3.79 |
| spectral power | 0.44 | 6.38 | 0.019 | 0.21 | -1.75 |
| bursts per second | 0.45 | 9.06 | 0.006 | 0.27 | **-3.95** |
| burst amplitude | 0.45 | 3.31 | 0.081 | 0.12 | 1.02 |

**Figure 2-6.** Model comparisons for predicting the rTE in Exp. 1 (N = 26). The ⍴ denotes Spearman's correlation coefficient. The F-statistic assesses whether the regression model to predict the rTE is better than a constant value, along with the corresponding p-value of the F-test. R^2^ represents the coefficient of determination, and lower values of the Akaike Information Criterion (AIC) indicate a better fit. Bold values indicate the best models, as determined by Spearman’s correlation and AIC. Note that the discrepancy of the best predictors according to Spearman’s correlation (non-parametric measures) and AIC (based on parametric models) can be explained by the differing robustness of the predictors: the relative burst time is more robust, whereas the number of bursts per second is more prone to segmentation artefacts (e.g., splitting a burst) depending on the threshold values described in the Methods section.
